# LASAGNA: A novel algorithm for transcription factor binding site alignment

**DOI:** 10.1186/1471-2105-14-108

**Published:** 2013-03-24

**Authors:** Chih Lee, Chun-Hsi Huang

**Affiliations:** 1 Department of Computer Science and Engineering, University of Connecticut, Fairfield Road, Storrs, CT 06269, USA

## Abstract

**Background:**

Scientists routinely scan DNA sequences for transcription factor (TF) binding sites (TFBSs). Most of the available tools rely on position-specific scoring matrices (PSSMs) constructed from aligned binding sites. Because of the resolutions of assays used to obtain TFBSs, databases such as TRANSFAC, ORegAnno and PAZAR store unaligned variable-length DNA segments containing binding sites of a TF. These DNA segments need to be aligned to build a PSSM. While the TRANSFAC database provides scoring matrices for TFs, nearly 78% of the TFs in the public release do not have matrices available. As work on TFBS alignment algorithms has been limited, it is highly desirable to have an alignment algorithm tailored to TFBSs.

**Results:**

We designed a novel algorithm named LASAGNA, which is aware of the lengths of input TFBSs and utilizes position dependence. Results on 189 TFs of 5 species in the TRANSFAC database showed that our method significantly outperformed ClustalW2 and MEME. We further compared a PSSM method dependent on LASAGNA to an alignment-free TFBS search method. Results on 89 TFs whose binding sites can be located in genomes showed that our method is significantly more precise at fixed recall rates. Finally, we described LASAGNA-ChIP, a more sophisticated version for ChIP (Chromatin immunoprecipitation) experiments. Under the one-per-sequence model, it showed comparable performance with MEME in discovering motifs in ChIP-seq peak sequences.

**Conclusions:**

We conclude that the LASAGNA algorithm is simple and effective in aligning variable-length binding sites. It has been integrated into a user-friendly webtool for TFBS search and visualization called LASAGNA-Search. The tool currently stores precomputed PSSM models for 189 TFs and 133 TFs built from TFBSs in the TRANSFAC Public database (release 7.0) and the ORegAnno database (08Nov10 dump), respectively. The webtool is available at http://biogrid.engr.uconn.edu/lasagna_search/.

## Background

A transcription factor is a protein that regulates the expression of its target genes by physically binding to the promoter regions of these genes. The binding sites of a transcription factor (TF) naturally share similarity with each other. The common pattern shared among the binding sites of a TF is called a motif. In general, there are two approaches to computational motif analysis, *de novo* motif discovery [1-12] and transcription factor binding site (TFBS) search [13-17]. As the name suggests, *de novo* motif discovery algorithms find over-represented patterns in sequences without prior knowledge of the binding TFs. The input to these algorithms is usually the upstream region sequences of genes putatively co-regulated by one or more common TFs. The output is one or more motifs or patterns whose instances are over-represented in the input sequences. On the other hand, a TFBS search algorithm takes binding site sequences of a TF as input. It learns from these known binding sites and builds a TF model out of them. The TF model can then be used to scan sequences for putative binding sites. While the two approaches are tightly connected, we focus on the TFBS search problem and assume that a TF has known binding sites available.

A typical TFBS search algorithm requires aligned TFBSs. This requirement allows simple representations of TF models. Types of TF models include consensus sequences, position-specific scoring matrices (PSSMs) [18], etc. The PSSM method is a widely used method among the available TFBS search algorithms. Given aligned binding sites of a TF, the TF model is essentially a 4×*l* matrix, where *l* is the length of the binding sites. Column *i* of the matrix stores the scores of matching the *i*^th^ letter in a sequence of length *l* (an *l*-mer) to nucleotides A, C, G and T, respectively. The score of an *l*-mer is then calculated by summing up the scores of letter 1 through letter *l*. Depending on the variant of PSSM, the score of A at position *i* can be the count of A at position *i* in the known TFBSs, the log-transformed probability of observing A at position *i*, or any other reasonable number. Once constructed, the matrix of a TF can be stored in a database to scan sequences for binding sites of the TF in the future without resorting to the actual binding sites. In fact, many tools [14,19-24] depend on matrices stored in at least one of the databases, JASPAR [25], RegulonDB [26] and TRANSFAC [27]. Since a matrix is constructed from aligned binding sites, we cannot overemphasize the quality of TFBS alignments.

Databases such as JASPAR, TRANSFAC and ORegAnno [28] contain DNA segments bound by TFs. These DNA segments can be seen as TFBSs with some irrelevant bases on one or both sides because of the resolutions of techniques used to obtain TFBSs. The DNA segments belonging to a TF are therefore unaligned variable-length sequences. While the DNA segments for most TFs in the JASPAR database are aligned, this is not the case for the TRANSFAC public and ORegAnno databases. About 53% (983 out of 1867) of the TFs in the TRANSFAC Public database (release 7.0) have unaligned variable-length DNA segments. Moreover, nearly 78% (1447 out of 1867) of TFs having curated DNA segments do not have a matrix. Focusing on TFs with variable-length DNA segments, about 71% (669 out of 983) of them do not have a matrix. On the other hand, the ORegAnno database stores experimentally validated DNA segments bound by TFs but does not provide matrices. About 31% (175 out of 572) of the TFs therein have variable-length DNA segments. In the absence of a matrix, to search for binding sites of these TFs using a matrix dependent tool, one needs to first align the curated DNA segments for each TF. In the rest of this paper, we refer to (variable-length) DNA segments containing TFBSs as (variable-length) TFBSs for simplicity reasons.

In this work, we propose a novel TFBS alignment algorithm named LASAGNA (Length-Aware Site Alignment Guided by Nucleotide Association). The algorithm is based on the hypothesis that binding sites of a TF share a core [29], a short and highly conserved stretch of DNA. Hence, a binding site can be seen as a core with some irrelevant bases on one or both sides. In general, shorter sites tend to contain fewer irrelevant bases and are easier to align. For this reason, we progressively align the binding sites from the shortest to the longest ones. The algorithm further exploits dependence between two positions in a binding site. Dependence between positions has been shown to boost performance of TFBS search algorithms [13,16] as well as protein structural motif recognition [30]. To our best knowledge, this idea has never been applied to multiple sequence alignment. We further describe a more sophisticated version, named LASAGNA-ChIP, for aligning peak sequences produced by ChIP-seq experiments.

To compare algorithms for TFBS alignment, we conduct cross-validation (CV) experiments on 4771 binding sites of 189 TFs across 5 species extracted from the TRANSFAC Public database (release 7.0). We compare LASAGNA to ClustalW2 [31,32] and MEME [1]. Being a widely used multiple sequence alignment algorithm, ClustalW2 was used to produce gapped TFBS alignments in creating the MAPPER database [33] as well as to produce both gapped and gapless TFBS alignments in [16]. ClustalW2 and other similar algorithms focus on producing structurally correct alignments, while other improved algorithms rely on structural or homology information [34]. ClustalW2 can be viewed as a representative of these algorithms when no information other than sequences is available. MEME, on the other hand, is a *de novo* motif discovery tool, whose input is typically regulatory regions of length 1,000 bp upstream of the genes presumably controlled by a common TF [35]. Nevertheless, a motif found in the input TFBSs can be used to align the TFBSs. In fact, MEME is employed by the PAZAR database [36] to dynamically align TFBSs and generate PSSMs. We show that LASAGNA significantly outperforms ClustalW2 (*p*-value: 1.22×10^−15^) and MEME (*p*-value: 3.55×10^−15^).

To scan promoters for new TFBSs based on variable-length known TFBSs, we couple a PSSM method with LASAGNA, denoted by LASAGNA-PSSM. That is, the input variable-length TFBSs are aligned by LASAGNA and a PSSM model is built from the alignment. It is useful to compare an alignment-based TFBS search method to an alignment-free method. Therefore, we further compare LASAGNA-PSSM to SiTaR [17], which accepts variable-length input TFBSs. To our best knowledge, SiTaR is the only alignment-free method capable of handling variable-length input TFBSs at the time of writing. Cross-validation results on 90 TFs whose binding sites can be located in respective genomes indicate that LASAGNA-PSSM is significantly more precise at fixed recall rates (*p*-value: 2.66×10^−8^). The recall-precision curve also shows that our method is constantly more precise at any recall rate and more sensitive at any precision.

Finally, we demonstrate the application of LASAGNA-ChIP to ChIP-seq data using 38 mouse ChIP-seq experiments. We show that, assuming the one-per-sequence model, LASAGNA-ChIP is comparable to MEME in revealing the motif of the ChIPed TF or its cofactor. For both LASAGNA-ChIP and MEME, the ChIPed TF motif was found in 31 experiments, while a cofactor motif was found in 3 experiments. While the two methods differ in the rest 4 experiments, the found motifs have similar information content and may belong to unknown cofactors.

## Methods

We describe our novel alignment algorithm in this section. LASAGNA utilizes a search module to align a new binding site with a partial alignment. Thus, we introduce the search module followed by the LASAGNA algorithm.

### The search module

The search module of LASAGNA is a function learned from a (partial) TFBS alignment to score *l*-mers. It considers nucleotide pairs in addition to individual nucleotides so as to exploit dependence between positions. We introduce our choice of the search module, the PSSM model described in [13]. We denote it by ${\text{PSSM}}_{K}^{a}(\cdot )$ in this work.

Suppose that a PSSM is constructed from aligned sequences of length *l*. The score of letter *u* at position *i* is given by

$$M_{i}(u)=\log\frac{f_{i}(u)}{f(u)},$$

where *f*_*i*_(*u*) is the probability of observing letter *u* at position *i* and *f*(*u*) is the background probability of seeing letter *u*. Similarly, the score of a pair of letters (*u*,*v*) at position (*i*,*j*) is given by

$$M_{i,j}(u,v)=\log\frac{f_{i,j}(u,v)}{f(u,v)},$$

where *f*_*i*,*j*_(*u*,*v*) is the probability of observing nucleotide pair (*u*,*v*) at position (*i*,*j*) and *f*(*u*,*v*) is the background probability of seeing the pair. The score of *s*, a sequence of length *l*, is then

(1) $${\text{PSSM}}_{K}(s)=\overset{l}{\underset{i=1}{\sum }}M_{i}(s_{i})+\overset{K}{\underset{k=1}{\sum }}\overset{l-k}{\underset{i=1}{\sum }}M_{i,j}(s_{i},s_{j}),$$

where *s*_*i*_ denotes the *i*^th^ letter of *s*, *j*=*i*+*k* and *K* is the scope parameter defined in [13]. The parameter *K* controls how far apart a pair of nucleotides can be. When *K*=1, only adjacent nucleotide pairs are scored. We define ${\text{PSSM}}_{0}(s)=\overset{l}{\underset{i=1}{\sum }}M_{i}(s_{i})$, that is, we do not score nucleotide pairs when *K*=0.

Our search module is a variant of (1). Let

$$M_{i}^{\prime }(u)=\left\{\begin{array}{ll} \underset{x}{\min}M_{i}(x) & \text{if}\;u\;\text{is the gap letter}\\ M_{i}(u) & \text{otherwise} \end{array}\right)$$

and

$$\;M_{i,j}^{\prime }(u,v)=\;\left\{\;\begin{array}{ll} \underset{x,y}{\min}M_{i,j}(x,y) & \;\;\text{if}\;u\;\mathrm{or}\;v\;\text{is the gap letter}\\ M_{i,j}(u,v) & \;\;\text{otherwise} \end{array}\right)\;.$$

The search module is defined as follows:

(2) $${\text{PSSM}}_{K}^{a}(s)=\overset{l}{\underset{i=1}{\sum }}M_{i}^{\prime }(s_{i})+\overset{K}{\underset{k=1}{\sum }}\overset{l-k}{\underset{i=1}{\sum }}M_{i,j}^{\prime }(s_{i},s_{j}),$$

where superscript a denotes alignment as this module is used in our alignment algorithm.

### The LASAGNA algorithm

The algorithm is based on the idea that the binding sites of a TF share a common core, a conserved short DNA sequence. A binding site can then be seen as a core with a few irrelevant bases on one or both sides. Assuming that each binding site fully contains the core, the shorter a binding site, the fewer irrelevant bases it contains. Therefore, we progressively align the binding sites by aligning the shortest binding site with the already aligned binding sites until all the binding sites are aligned.

The algorithm takes a set of unaligned binding sites, *U*, and parameter *K*_a_ as inputs. Let *A* denote the set of aligned binding sites. A binding site in *A* may have gap letters added to one or both ends as a result of the alignment. The algorithm works as follows: 

1. Initialize *A* to {*s*}, where *s*, the *seed site*, a shortest binding site arbitrarily chosen from *U*. Remove *s* from *U*.

2. 

(a) Build ${\text{PSSM}}_{K_{a}}^{a}(\cdot )$ from *A*. Let the length of this PSSM be *l*.

(a) Remove the shortest binding site *s* from *U*.

(a) Create *S*, the augmented sequence of *s*, by adding *l*−1 gap letters to both ends of *s*.

(a) Score each *l*-mer of *S* by ${\text{PSSM}}_{K_{a}}^{a}(\cdot )$ to find the highest scoring one.

(a) Let *s* be its reverse-complement and repeat c–d. That is, the opposite strand is considered.

3. Add *s* to *A* if the highest scoring *l*-mer resides in *s*. Otherwise, add its reverse-complement to *A*. Gap letters are added to one or both ends of sequences in *A*. This ensures that they are all of the same length and each column of the alignment has at least one non-gap letter.

4. Repeat 2–3 until *U* is empty.

**Figure 1:**
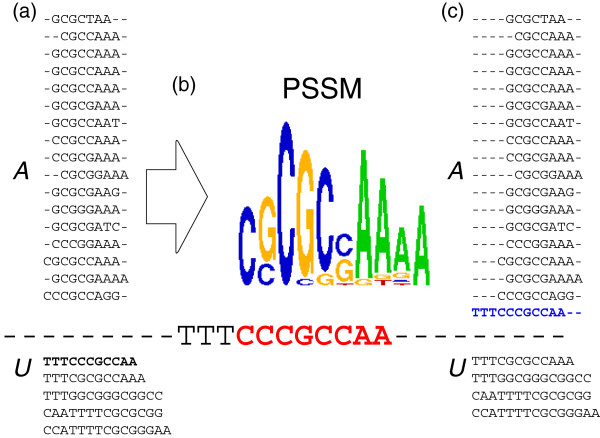
**An illustration of LASAGNA with *K***_***a ***_***=0 *.** (**a**) The aligned binding sites in *A* and the unaligned ones in *U*. The shortest binding site is in bold. (**b**) The sequence logo [48] of the PSSM built from *A* aligns with the augmented sequence - - - - - - - - - TTTCCCGCCAA - - - - - - - - -, where the matched portion is in bold. (**c**) The updated *A* and *U*, where the newly added binding site is in bold.

In step 2b, there may be more than one shortest binding sites in *U*. To break the tie, we use ${\text{PSSM}}_{K_{a}}^{a}(\cdot )$ to scan each of the shortest ones. The “*s*” containing the highest scoring *l*-mer is removed from *U* to align with sequences in *A*. In the unlikely case of two or more shortest binding sites in *U* sharing the same highest score, one is arbitrarily chosen. Figure 1 illustrates an iteration of the algorithm.

An alignment may be trimmed before building a PSSM. We describe one way of trimming aligned TF binding sites using two simple measures. Let *l* be the length of the aligned binding sites. We first compute and denote the percentage of non-gap letters at position *i* of the alignment by *C*_*i*_, for *i*=1,2,…,*l*. The information content (IC) at each position is then computed with small sample correction described in [37]. That is,

$$\;IC_{i}=\max\left\{0,\;\;\;2+\underset{u\in \{\text{A, C, G, T}\}}{\sum }f_{i}(u)\underset{2}{\log}f_{i}(u)-ê(n_{i})\right\},$$

where *i*∈{1,2,…,*l*}, *n*_*i*_ is the number of non-gap letters at position *i* and ê(·) gives the approximated sampling error. Let *C*_min_ and *IC*_min_ be the cutoff thresholds. The alignment is examined from the left end to the right until the first position *j* satisfying both *C*_*j*_>*C*_min_ and *IC*_*j*_>*IC*_min_ is encountered. The positions preceding *j* are trimmed off. The trimming is similarly applied to the right end.

### LASAGNA for ChIP-seq data

Although LASAGNA is not specifically designed as a *de novo* motif discovery algorithm, a more sophisticated version, named LASAGNA-ChIP, is capable of handling ChIP-seq data. Here, we refer to the previous section and describe the additional steps that are necessary for aligning ChIP-seq peak sequences. The flowchart in Additional file 1 gives an overview of LASAGNA-ChIP.

Before aligning ChIP-seq peak sequences, each sequence is clipped to 100 bp surrounding the signal peak. This is a common practice since, for most peak sequences (>90%), the actual TFBS is usually found within 50 bp of the called peak [38]. In step 2a, we trim the partial alignment *A* if it contains more than two sequences. Unlike TFBSs found in databases such as TRANSFAC, even after clipping, a peak sequence contains much more irrelevant bases flanking the core. The trimming procedure described in the previous section is used, where *C*_min_ (*IC*_min_) is set to the mean *C*_*i*_ (*IC*_*i*_) over all the columns of *A*. The resulting alignment is further trimmed by IC such that it has at most 15 columns and the columns on both ends have positive IC. In step 2b, if there are more than 5 shortest binding sites in *U*. Only 5 are arbitrarily chosen to break the tie by similarity to ${\text{PSSM}}_{K_{a}}^{a}(\cdot )$.

The alignment *A* obtained by the modified procedure is further refined as follows: 

1. Set *T* to *A* trimmed to *l* columns as described above.

2. Build ${\text{PSSM}}_{K_{a}}^{a}(\cdot )$ out of *T*.

3. Initialize *R* to {}, the refined partial alignment.

4. For each peak sequence *s*, 

(a) Create *S*, the augmented sequence of *s*, by adding *l*−1 gap letters to both ends of *s*.

(a) Score each *l*-mer of *S* by ${\text{PSSM}}_{K_{a}}^{a}(\cdot )$ to find the highest scoring one.

(a) Let *s* be its reverse-complement and repeat a–b.

(a) Add *s* to *R* if the highest scoring *l*-mer resides in *s*. Otherwise, add its reverse-complement to *R*. Gap letters are added to one or both ends of sequences in *R*.

5. Set *A* to *R* and repeat 1–5 until the sum of IC across columns of *T* does not change in 3 iterations.

For ChIP-seq peak sequences, the shortest sequence may miss or contain only a fraction of the core. Hence, using the shortest sequence as the *seed site* sometimes results in an alignment with less IC. For this reason, five additional sequences are arbitrarily chosen as the *seed site* to produce 5 additional alignments. Among the 6 alignments, the one with the most IC after trimming is chosen as the final alignment.

### Scoring a putative binding site

Although a PSSM suggests the length of a putative binding site, we do not restrict the length of a candidate binding site to the length of the PSSM. A putative binding site could be of any reasonable length. If a true binding site is flanked by a few irrelevant bases, this sequence should be given a relatively high score compared to those of non-binding sites. Therefore, to score a putative binding site *s*, we slide *s* through the PSSM as described in the previous section. The score of sequence *s* is given by

(3) $${\text{Score}}_{K_{s}}(s)=\underset{i\in \{1,2,\ldots ,l+l_{s}-1\}}{\max}{\text{PSSM}}_{K_{s}}(S_{i:(i+l-1)}),$$

where *l* is the length of the PSSM, *l*_*s*_ is the length of *s*, *S* denotes the augmented sequence of *s* with *l*−1 gap letters on both ends and ${\text{PSSM}}_{K_{s}}(\cdot )$ is defined in (1).

## Results and discussion

### Comparison of alignment algorithms

#### Data sets

We downloaded all the TF binding sites from the TRANSFAC Public database (release 7.0). The binding sites were grouped by species and TF. Binding sites having less than 4 nucleotides were discarded. TFs of each species were filtered such that each TF has at least 10 binding sites. This ensures that each TF has enough binding sites to construct a PSSM. The numbers of TFs and TFBSs are listed in Table 1.

**Table 1:** TFBSs in TRANSFAC public database by species

**Species**	**# TFs**^***1***^	**# TFBSs**^***2***^
Homo sapiens	68	1984
Mus musculus	53	966
Rattus norvegicus	26	633
Drosophila melanogaster	29	935
Saccharomyces cerevisiae	13	253
Overall	189	4771

1. The total number of TFs.

2. The total number of TFBSs.

To facilitate experiments, we planted each TFBS in a 2000 base random sequence simulated by a first-order Markov chain of the species in question. Except for Saccharomyces cerevisiae, the Markov chain of a species was learned from promoter sequences in the UCSC Genome Browser database [39]. For Saccharomyces cerevisiae, the promoter sequences were retrieved from the SCPD [40] using the yeast gene list in euGenes [41].

#### Performance assessment and evaluation metrics

Since the purpose of aligning TFBSs is to construct a PSSM, the quality of an alignment is best measured by the search performance of the PSSM. The performance of a TFBS search method is evaluated by *ν*-fold CV. Consider a TF with *n* binding sites. The *n* TFBSs are first divided into *ν* sets, each of which contains $\left\lfloor \frac{n}{\nu }\right\rfloor$ or $\left\lfloor \frac{n}{\nu }\right\rfloor$ + 1 TFBSs. At each iteration of the *ν*-fold CV, one of the *ν* TFBS sets called the *test TFBS set*, *P*_test_, is left out. The rest of the TFBSs are aligned to build a PSSM. Each test TFBS in *P*_test_ is then planted in a 2000 base random sequence and scanned by the PSSM, scoring each *l*-mer, where *l* is the length of the test TFBS. We score both the forward and reverse strands of an *l*-mer and assign the higher score to it. An *l*-mer is considered a hit if it shares more than ⌊*l*/2⌋ bases with the test TFBS. The *l*-mers can then be divided into two sets, *H* and *N*, where *H* is the set of hits and *N* is considered the set of non-binding sites. The score of the test TFBS is the highest score of hits in *H*. For each test TFBS *t*∈*P*_test_, we find its rank relative to all the non-binding sites in *N*. Formally, the rank of binding site *t* equals $1+|\{s\in N|{\text{Score}}_{K_{s}}(s)\geq {\text{Score}}_{K_{s}}(t)\}|$.

After the *ν*-fold CV, we end up with *n* ranks, each of which corresponds to a TFBS. We use the area under the ROC curve (AUC) to gauge the quality of alignment. The ROC curve is a plot of true positive rate (TPR) against false positive rate (FPR), displaying the trade-off between TPR and FPR. We refer readers to [42] for an introduction to this metric. In this study, *ν*=10 for all the CV experiments.

#### Comparison with ClustalW2

In general, gapless alignment is preferred over gapped alignment for aligning TFBSs. Because of the nature of ClustalW2, the alignment of TFBSs may contain gaps in the middle of some binding sites. This is disadvantageous to ClustalW2 as the PSSM method does not allow insertion of gaps into the sequence being scanned. Hence, we turned off gaps by setting the gap opening penalty parameters to a large value, i.e., we set both GAPOPEN and PWGAPOPEN to 100000. Indeed, results indicated that overall the “gapped” ClustalW2 performs slightly worse than the “gapless” variant (*p*-value: 0.277). For both LASAGNA and ClustalW2, parameter *K*_s_ in Eq. 3 was searched from 0 to min{10,*l*_min_−1} for each TF and the one producing the highest AUC is used, where *l*_min_ is the minimal length of the TFBSs. For LASAGNA, parameter *K*_a_ of the LASAGNA algorithm was set to *K*_s_ as the two parameters are closely related.

**Figure 2:**
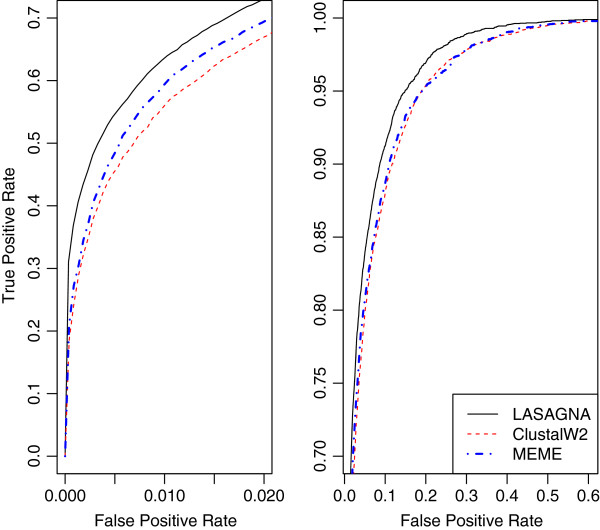
**Overall ROC curves for the three alignment algorithms.** The left panel shows the curves at low false positive rates, from 0 to 0.02. The right panel presents the curves at false positive rates from 0.02 to 0.6. The three methods are indistinguishable when the false positive rate is greater than 0.6 and hence the region is not shown. We note that the vertical axes of the two panels are on different scales.

We conducted 10-fold CV on each TF. The overall ROC curves are shown in Figure 2. The ROC curves are based on the ranks of 4771 TFBSs of 189 TFs. It shows that LASAGNA has invariably higher true positive rate than ClustalW2. The AUC score was calculated for each TF and for each method. To gauge the significance of difference, the Wilcoxon signed-rank test [43] was performed for each species. The tests showed that LASAGNA is consistently better than ClustalW2 across the 5 species. Table 2 shows the test results. Overall, LASAGNA performed significantly better than ClustalW2 in terms of AUC scores. The species-wise *p*-values shows that LASAGNA is significantly better (<0.05) than ClustalW2 for aligning TFBSs of all the 5 individual species.

**Table 2:** Species-wise and overall comparisons between LASAGNA and ClustalW2

**Species**	**# better**^***1***^	**# ties**^***2***^	**# TFs**^***3***^	***p*-value**^***4***^
H. sapiens	54 (79.4%)	0	68	4.42×10^−7^
M. musculus	42 (79.2%)	0	53	1.41×10^−5^
D. melanogaster	22 (75.9%)	0	29	9.89×10^−4^
S. cerevisiae	9 (69.2%)	1	13	3.88×10^−2^
R. norvegicus	20 (76.9%)	1	26	1.54×10^−3^
Overall	147 (77.8%)	2	189	1.22×10^−15^

1. Number of TFs on which LASAGNA performs better than ClustalW2.

2. Number of TFs on which LASAGNA and ClustalW2 have the same performance.

3. Total number of TFs for a species.

4. Wilcoxon signed-rank test *p*-value.

 To better understand the results, we split the 189 TFs into two groups. One contains TFs on which LASAGNA performed better than ClustalW2 and the other contains the rest of the TFs. Three factors are examined for each TF. They are the number of TFBSs, the mean and standard deviation of TFBS length. For each factor, we looked for difference between the two groups. Table 3 shows the comparisons. It can be seen that LASAGNA produces better alignments when a TF has fewer binding sites but the difference is not significant. The mean and standard deviation of TFBS length are the two more important factors. We believe that LASAGNA is well-suited for aligning TFBSs that are longer and more variable in length.

#### Comparison with MEME

The MEME tool in the MEME Suite 4.8.1 was used. The parameter minw, minimal width of motifs, was set to the smaller of 6 and the minimal length of input TFBSs. The option revcomp to search the reverse strand was turned on. Finally, the parameter minsites was set to the number of input TFBSs since a common motif is supposed to appear at least once in each TFBS. To ensure that MEME functions properly, binding sites shorter than 8 bases are padded with gap letters since genomic locations are not available for most TFBSs.

**Table 3:** Comparison of two groups of TFs divided according to results on LASAGNA and ClustalW2

**Factor**	**Group 1**^***1***^**mean**	**Group 2**^***2***^**mean**	***p*-value**^***3***^
# TFBSs ^4^	25.07483	25.83333	0.1409
Mean of TFBS length	18.78626	17.56167	0.08451
SD of TFBS length ^5^	8.180204	6.921905	0.06295

1. LASAGNA performed better than ClustalW2 on TFs in this group.

2. ClustalW2 performed better than or equal to LASAGNA on TFs in this group.

3. Wilcoxon signed-rank test *p*-value.

4. Number of binding sites for each TF.

5. Standard deviation of binding site length for each TF.

The experiments were carried out in the same manner as the ClustalW2 experiments. The overall ROC curve in Figure 2 indicates that LASAGNA has consistently higher true positive rates than MEME across different false positive rates. The overall and species-wise comparisons between LASAGNA and MEME in Table 4 show that LASAGNA performed significantly better than MEME. To gain some insights into the difference between LASAGNA and MEME, we similarly examined the three factors used to compare LASAGNA and ClustalW2. As seen in Table 5, the number of input TFBSs is the only significant (*p*-value < 0.05) factor out of the three. The reasons are not clear but may be investigated in the future. Moreover, it will be helpful to identify other (biologically meaningful) factors that can better explain the performance difference.

**Table 4:** Species-wise and overall comparisons between LASAGNA and MEME

**Species**	**# better**^***1***^	**# ties**^***2***^	**# TFs**^***3***^	***p*-value**^***4***^
H. sapiens	41 (60.3%)	0	68	7.83×10^−3^
M. musculus	41 (77.4%)	0	53	8.79×10^−6^
D. melanogaster	26 (89.7%)	0	29	1.02×10^−7^
S. cerevisiae	10 (76.9%)	3	13	2.96×10^−3^
R. norvegicus	23 (88.5%)	1	26	1.73×10^−4^
Overall	141 (74.6%)	4	189	3.55×10^−15^

1. Number of TFs on which LASAGNA performs better than MEME.

2. Number of TFs on which LASAGNA and MEME have the same performance.

3. Total number of TFs for a species.

4. Wilcoxon signed-rank test *p*-value.

**Table 5:** Comparison of two groups of TFs divided according to results on LASAGNA and MEME

**Factor**	**Group 1**^***1***^**mean**	**Group 2**^***2***^**mean**	***p*-value**^***3***^
# TFBSs^4^	23.33333	30.85417	0.03196
Mean of TFBS length	18.33468	19.04125	0.3007
SD of TFBS length ^5^	7.95844	7.730625	0.1846

1. LASAGNA performed better than MEME on TFs in this group.

2. MEME performed better than or equal to LASAGNA on TFs in this group.

3. Wilcoxon signed-rank test *p*-value.

4. Number of binding sites for each TF.

5. Standard deviation of binding site length for each TF.

#### Distribution of *K*_s_

In Additional file 2, for LASAGNA, ClustalW2 and MEME, we show the distribution of *K*_s_ for a TF by species and conserved domain. Overall, we observe that small values are preferred for all three methods. By visual inspection, LASAGNA appears more similar to MEME than ClustalW2 in the usage of *K*_s_. It can be seen that the usage of *K*_s_ differs among different conserved domains. Related conserved domains such as ZF-H2C2_2 and ZF-C2H2 display similar patterns. This is not surprising as conserved domains in a protein are often computationally predicted. Hence, a protein is likely to possess related conserved domains. While overall the distributions seem method-dependent, we observe that, for ZF-H2C2_2 and ZF-C2H2, the distributions center around 4 across all three methods. Finally, we note that these observations are preliminary and more TFs are needed to draw statistically sound conclusions.

### Comparison of TFBS search methods

#### Data sets

To compare with an alignment-free TFBS search method, SiTaR, [17], we retrieved real promoter sequences embedding TFBSs. Specifically, we followed the curated location of each binding site in the TRANSFAC Public database (release 7.0) to retrieve the 1000-base sequences flanking the binding site. We discarded binding sites that cannot be found in the proximity of the curated locations. The retrieved binding sites were grouped by TF and TFs having less than 10 binding sites were removed. After filtering, we ended up with 90 TFs and 1751 binding sites. A TF may be present in more than one species as homologs and hence the binding sites of a TF may be located in genomes of multiple species. The species and respective numbers of binding sites are shown in Table 6.

**Table 6:** Distribution of the 1751 binding sites of 90 TFs in TRANSFAC public database

**Species**	**# TFBSs**^**1**^
Homo sapiens	735
Mus musculus	346
Rattus norvegicus	278
Saccharomyces cerevisiae	158
Drosophila melanogaster	155
Gallus gallus	73
Bos taurus	5
Sus scrofa	1

1. Total number of TFBSs.

#### Performance assessment and evaluation metrics

To compare with SiTaR [17], we adopt the same *ν*-fold CV process used to compare LASAGNA with ClustalW2 and MEME. However, we do not assume that a TFBS search method scores all the *l*-mers in a promoter sequence, where *l* is the length of binding sites. Instead, a TFBS search method scans a promoter sequence and predicts a list of binding sites with respective scores. The predicted binding sites may be of different lengths, which is the case for SiTaR.

We describe how a hit is determined. Let the length of a predicted binding site be *l* and the length of the test TFBS in question be *l*_s_. The predicted binding site is considered a hit to the test TFBS if the overlap between the two sequences is more than ⌊*l*_s_/2⌋ bases as in [17]. In case this is not possible, i.e., *l*≤⌊*l*_s_/2⌋, the predicted binding site must be embedded in the true one to be deemed a hit.

Using the *n* ranks of TFBSs from *ν*-fold CV, we compute recall (true positive rate), precision and the *F*_*β*_-measure, where *β*=0.5 as in [17]. Let the recall rate be *r*. The number of TFBSs recalled by the method is *p*_T_=*n*×*r*. Let the number of non-binding sites or false positives introduced be *p*_F_. The precision is given by $\frac{p_{T}}{p_{T}+p_{F}}$, while $F_{\beta }=(1+\beta^{2})\frac{\text{precision}\times \text{recall}}{\beta^{2}\times \text{precision}+\text{recall}}$.

#### Comparison with an alignment-free method

We conducted 10-fold CV on the aforementioned 90 TFs. The PSSM method dependent on LASAGNA (LASAGNA-PSSM) was compared to SiTaR [17]. LASAGNA considered both strands of a sequence when aligning binding sites. The parameters *K*_a_=*K*_s_ were determined in the same way as in comparing LASAGNA to ClustalW2. An alignment was trimmed with *C*_min_=0.4 and *IC*_min_=0 before constructing a PSSM as described in the method section on the LASAGNA algorithm. The PSSM method uses a cutoff score to predict TFBSs. The cutoff score is set to the minimal score of the constituting binding sites of the PSSM. The SiTaR method has a mismatch parameter and the maximal value allowed by its webtool is 5. We searched in the range from 0 to 5 to find the mismatch value giving the highest *F*_*β*_-measure for each TF.

**Figure 3:**
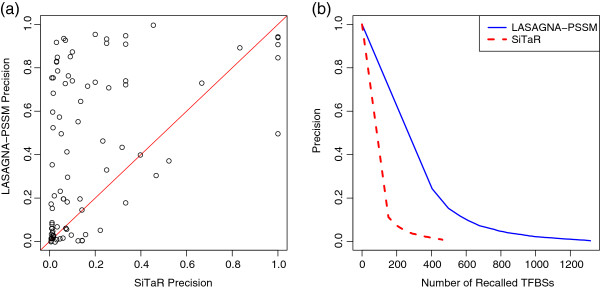
**Comparison of the PSSM method dependent on LASAGNA to SiTaR.** (**a**) Scatter plot of precision by LASAGNA-PSSM against precision by SiTaR at the same recall rate for each TF. Each point corresponds to a TF. Seventy-three percent (65 out of 89) of the TFs are above the reference line, indicating that LASAGNA-PSSM is more precise for the 65 TFs. (**b**) Plots of precision against recall for LASAGNA-PSSM and SiTaR based on all the 90 TFs.

In terms of the F _*β*_, no significant difference was found between the two methods (*p*-value: 0.392 [43]). To ensure a fair comparison, we fixed the recall rate for each TF and compare the precision achieved by LASAGNA-PSSM and SiTaR. The recall rate was set to the lower of the recall rates attained by LASAGNA-PSSM and SiTaR. The TF c-Jun (AC: T00132) was excluded from comparison because SiTaR did not recover any TFBS. Figure 3a shows the plot of precision by LASAGNA-PSSM against that by SiTaR. At fixed recall rates, LASAGNA-PSSM is more precise than SiTaR on 65 out of 89 TFs (*p*-value: 2.66×10^−8^). Figure 3b shows the plots of precision against recall based on all the recalled TFBSs by each method. It can be seen that LASAGNA-PSSM is constantly more precise than SiTaR at the same recall rate. Moreover, LASAGNA-PSSM recovered substantially more TFBSs than SiTaR at the same precision.

Results reported in [17] showed that SiTaR is highly precise and sensitive. Although SiTaR accepts variable-length binding sites, all the experiments presented in [17] used fixed-length binding sites as inputs. It is therefore not clear how SiTaR performs on TFs having variable-length binding sites. It is also not clear whether SiTaR preprocesses highly variable-length binding sites as this was not stated in [17]. These issues however are not the focus of our work.

### Application of LASAGNA-ChIP to ChIP-seq data

To demonstrate the use of LASAGNA-ChIP on ChIP-seq data, we retrieved mouse ChIP-seq data produced by the Encyclopedia of DNA Elements (ENCODE) project [44] from the UCSC Genome Browser [39]. All the 38 peak files in the Narrow Peaks format that matches pattern http://hgdownload.cse.ucsc.edu/goldenPath/mm9/database/wgEncode*Tfbs*Pk*were downloaded on Oct. 12, 2012, where “*” is the wildcard character matching zero or more characters. These files give signal peak location besides start and end for each peak and hence the corresponding signal files do not need to be processed by a peak-finding algorithm. Four distinct cell types and 17 distinct target TFs are present in the 38 ChIP-seq experiments. Additional file 3 lists, for each ChIP-seq experiment, the cell, target TF, number of peaks as well as the minimum, maximum, mean and standard deviation of peak length. We observe that the peak length varies greatly. The mean peak length can be as long as 1124, while the highest standard deviation is nearly 876.

It is useful to know if LASAGNA-ChIP is able to align peak sequences and reveal the motif of the ChIPed TF. To align peak sequences, parameter *K*_a_ was searched from 0 to 8 to obtain the alignment with the highest IC. MEME was also used to align peak sequences because it is often the choice of method. In fact, MEME is used by 5 out of 6 tools compared in [45] for ChIP-seq data analysis. The MEME parameters are described in section Comparison of alignment algorithms, where the one-per-sequence model is assumed. To ensure that both methods finish within reasonable time, for each experiment, we randomly sampled 300 peaks for alignment. We did not distinguish large peaks from small ones because ChIP-seq experiments require large numbers of cells and hence “a small peak could represent very strong binding in only a subset of the cells” [46].

For each alignment, we searched for the resulting motif in 386 UniPROBE mouse motifs and 398 motifs derived from all the matrices in the TRANSFAC Public database. The search was accomplished by software TOMTOM [47]. We used Pearson correlation as the distance measure, required a minimal overlap of 5 nucleotides, and set the E-value cut-off to 5. Additional file 4 shows, for each ChIP-seq experiment, the sequence logos of motifs found by LASAGNA-ChIP and MEME. The matching motifs found by TOMTOM are listed under each sequence logo [48] by E-value. In case more than 10 significant motifs were found, only the 10 most significant ones were shown. The one matching the ChIPed TF is highlighted in yellow for each ChIP-seq experiment.

We first notice that overall the motifs found by LASAGNA-ChIP and MEME are very similar by visual inspection. No significant difference is observed in terms of motif IC (*p*-value: 0.1252). For both LASAGNA-ChIP and MEME, the ChIPed TF motifs were found for 31 experiments. Among the other 7 experiments are one MYB in MEL cells, all the ETS1 in CH12 and MEL cells, one JUND in MEL cells, one MAX in C2C12 cells, all the TBP in CH12 and MEL cells. Interestingly, LASAGNA-ChIP and MEME differ only for 4 out of these 7 experiments. They are one ETS1 in CH12 cells, one MAX in C2C12 cells and two TBP in CH12 and MEL cells. Although LASAGNA-ChIP and MEME differ in these cases, the found motifs still warrant further analyses. For instance, the motif for ETS1 in CH12 cells found by LASAGNA-ChIP resembles the secondary motif of Gabpa, which is a known paralog.

For the other 3 out of the 7 experiments, LASAGNA-ChIP and MEME produced similar motifs. The one found for MYB in MEL resembles those of GATA proteins. This agrees with a recent study reporting that MYB and GATA-3 cooperatively regulate IL-13 by direct binding to a conserved GATA-3 response element [49]. Since this motif is based on 300 peak sequences, it is likely that the two proteins similarly regulate other genes in MEL cells. The motif for ETS1 in MEL cells also matches those of GATA proteins. Cooperation between ETS1 and GATA-3 in regulating IL-5 was also suggested [50,51]. Finally, while the motif for JUND in MEL cells matches two motifs in the TRANSFAC and UniPROBE databases, the matches are likely false positives since no literature support was found.

While it is not specifically designed to be a *de novo* motif discovery method, LASAGNA-ChIP aligns all the peak sequences and finds the most informative motif. The assumption that a motif instance is present in each peak sequence may not hold for some experiments. Because of several possible binding models [46], two or more motifs may be present in subsets of the peak sequences. Discovery of more than one motif will be enabled for LASAGNA-ChIP in the near future.

### LASAGNA is simple and effective

Unlike MEME and similar methods, the order in which the input sequences are aligned is crucial to LASAGNA and ClustalW. ClustalW relies on a guide tree based on pairwise alignments to decide the order. LASAGNA, on the other hand, depends on the length of a sequence and its similarity to the partial alignment. LASAGNA-ChIP is well-suited for a TF whose shortest site misses the core or contains only a fraction of it. We, however, observed no significant difference between LASAGNA and LASAGNA-ChIP on TFBSs in the TRANSFAC Public database. This is because, for these TFBSs, a shortest site often fully contains the core.Hence, our assumption holds true in general.

For ChIP-seq data, the assumption that short sequences contain less irrelevant bases flanking the core may not hold. However, we observe that, under the one-per-sequence model, LASAGNA-ChIP performed comparably well to MEME in aligning ChIP-seq peak sequences. We attempted other orders such as from the longest sequence to the shortest one and found that aligning the shortest sequence first does have its advantage (data not shown). Also, we note that, for 11 out of 38 experiments, the peak sequences are all at least 100 bp (see Additional file 3) and hence all the peak sequences are 100 bp long after clipping. This implies that LASAGNA-ChIP is capable of handling sequences of the same length.

LASAGNA-ChIP, MEME and methods alike produce gapless alignments and do have their limits. When a TF binds to two cores separated by a variable-length spacer, these methods are expected to align the canonical TFBSs containing spacers of the most prevalent length. These binding patterns are also known as two-block motifs. Gapped alignment or explicit modeling [52] is needed to correctly align TFBSs of this nature.

### Implementation

We have implemented a user-friendly webtool named LASAGNA-Search, which is freely available at http://biogrid.engr.uconn.edu/lasagna_search/. Useful features include automatic promoter retrieval, visualization of hits locally and at the UCSC Genome Browser, and automatic gene regulatory network construction based on significant hits. LASAGNA-Search adopts the LASAGNA-PSSM method and currently stores PSSM ${}_{K_{s}}$ models (PSSM models for short), where *K*_s_ is determined by CV experiments, for the 189 TRANSFAC TFs summarized in Table 1 as well as 133 TFs from ORegAnno (08Nov10 dump). In Additional file 5, we list each model with its counterpart for the same TF if one is found in matrices in TRANSFAC Public. We do not evaluate models by IC because higher IC implies higher specificity but not necessarily higher sensitivity. Comparison with models in other databases is beyond the scope of this study but will be investigated in the near future.

LASAGNA-Search estimates *p*-values of PSSM scores empirically because the PSSM model for a TF may score nucleotide pairs in addition to individual nucleotides. When *K*_s_=0, a PSSM score is considered the sum of independent variables and hence the exact *p*-value can be efficiently computed [53]. Even with this independence assumption, the scores of nucleotide pairs at (1, 2) and (2, 3), for instance, are never independent. Hence, a PSSM score cannot be seen as the sum of independent variables when nucleotide pairs are scored. The empirical PSSM score distribution of a TF is obtained from scanning a random sequence simulated by *f*(*u*),*u*∈{A, C, G, T}, where *f*(*u*) is estimated from all the TFBSs used to build the PSSM. LASAGNA-Search focuses on only PSSM scores in the upper 5% and hence scores in the lower 95% are given a *p*-value of 0.05+. Currently, the smallest nonzero *p*-value is 2.5×10^−5^ and 0 means any number less than 2.5×10^−5^.

**Figure 4:**
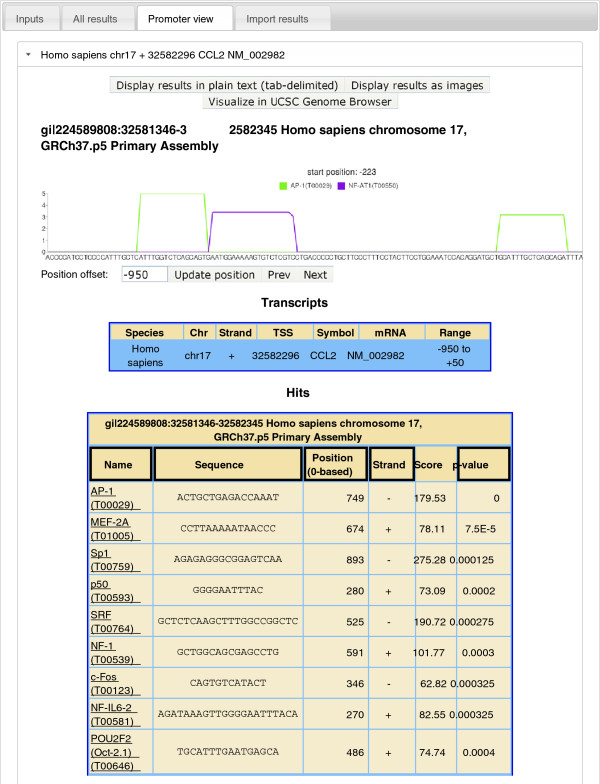
**Partial results of scanning the promoter of human gene CCL2.** The list of predicted binding sites are sorted by *p*-value in ascending order while only the top-4 hits are shown. The best hit is visualized in the context of other binding sites over a stretch of the promoter, where the height of a box is − log10*p*-value. CCL2 is known to be a target gene of AP-1, Sp1 and p50 [28]. These 3 binding sites are not in the TRANSFAC public database and were not used to build the PSSMs.

As a case study, we scanned the promoter region of human gene CCL2 (NCBI Gene ID 6347), also known as MCP1. CCL2 was arbitrarily chosen by browsing the ORegAnno database [28]. The promoter sequence (-950 to +50 relative to the transcription start site) was automatically retrieved and scanned for binding sites of all 68 human TFs with the *p*-value threshold set to 0.001. Figure 4 displays a partial view of the search results ordered by *p*-value. The only 3 true positive hits, AP-1, Sp1 and p50 (NFKB1), were found in the top-4 of the list. According to ORegAnno, CCL2 is a target gene of AP-1, Sp1, NFKB1, STAT1 and GAS, where GAS likely refers to the gamma activated site bound by STAT1. STAT1, however, is not one of the 68 TFs and hence all the TFs known to regulate CCL2 were recalled. The fact that AP-1, Sp1 and p50 regulate CCL2 is also documented in TRANSFAC [27] (T00029, T00759 and T00593). The actual sites (R14639, R14638 and R14640), however, are not in the public release and were not used to build the PSSM models. We note that this case study is for illustration not evaluation purposes.

## Conclusions

We proposed LASAGNA, a novel alignment algorithm specifically designed for aligning variable-length transcription factor binding sites. Cross-validation results on 189 TFs and 4771 TFBSs indicated that LASAGNA significantly outperformed ClustalW2 (*p*-value: 1.22×10^−15^) and MEME (*p*-value: 3.55×10^−15^). This is because LASAGNA was specifically designed for aligning variable-length TFBSs. Based on the success of LASAGNA, we developed LASAGNA-ChIP, which is capable of handling sequences produced by ChIP-chip and ChIP-seq experiments. While ClustalW2 is better suited for producing structurally correct alignments, LASAGNA-ChIP, MEME and methods alike can be used to align sequences produced by ChIP-chip or ChIP-seq experiments.

We compared LASAGNA-PSSM, the PSSM method dependent on LASAGNA, to SiTaR, an alignment free TFBS search method. Cross-validation experiments were conducted on 1751 TFBSs of 90 TFs for both methods. The results showed that, at fixed recall rates, LASAGNA-PSSM is significantly more precise than SiTaR (*p*-value: 2.66×10^−8^). The recall-precision curve showed that our method is constantly more precise at any recall rate or more sensitive at any precision.

We conclude that the LASAGNA algorithm is simple and effective in aligning variable-length binding sites. It has been integrated into a user-friendly webtool for TFBS search called LASAGNA-Search. The tool currently stores precomputed PSSM models for 189 TFs and 133 TFs built from TFBSs in the TRANSFAC Public database (release 7.0) and the ORegAnno database (08Nov10 dump), respectively. In the future, more sources of experimentally validated TFBSs such as the PAZAR database will be incorporated into the webtool, making variable-length TFBSs more accessible to scientists in the field.

## Competing interests

Both authors declare that they have no competing interests.

## Authors’ contributions

CH conceived the study. CL collected the data, carried out the experiments and drafted the manuscript. CH guided the study and revised the manuscript. Both authors read and approved the final manuscript.

## Supplementary Material

Additional file 1 LASAGNA-ChIP flowchart. Click here for file

Additional file 2 **Distribution of *K***_***s***_** by species and conserved domain.** Click here for file

Additional file 3 **Summary of 38 mouse ChIP-seq experiments.** Each row shows the track name in the UCSC Genome Browser, cell type, target TF, number of peak sequences as well as the minimum, maximum, mean and standard deviation of peak sequence length. Click here for file

Additional file 4 **Motifs found by LASAGNA-ChIP and MEME.** For each ChIP-seq experiment, the sequence logos of motifs found by LASAGNA-ChIP and MEME are shown. The matching motifs in the TRANSFAC Public and UniPROBE databases found by TOMTOM are listed below each sequence logo. The first ChIPed motif TF is highlighted in yellow if it is among the matching motifs. When the found motif does not resemble those of the ChIPed TF, the first cofactor of the ChIPed TF is highlighted in blue if it is among the matching motifs. Other possibly correct matches are highlighted in green. Click here for file

Additional file 5 **List of LASAGNA-built models based on TRANSFAC/ORegAnno TFBSs.** Only models whose counterparts can be found in matrices in TRANSFAC Public are listed. The IC and number of sites are shown for each model. Click here for file
