# Supplementary figures and images for: LASAGNA: A novel algorithm for transcription factor binding site alignment
Source: BMC Bioinformatics. 2013 Mar 24;14:108. doi: 10.1186/1471-2105-14-108 (PMC3747862; doi:10.1186/1471-2105-14-108)

Overview of the LASAGNA-ChIP algorithm

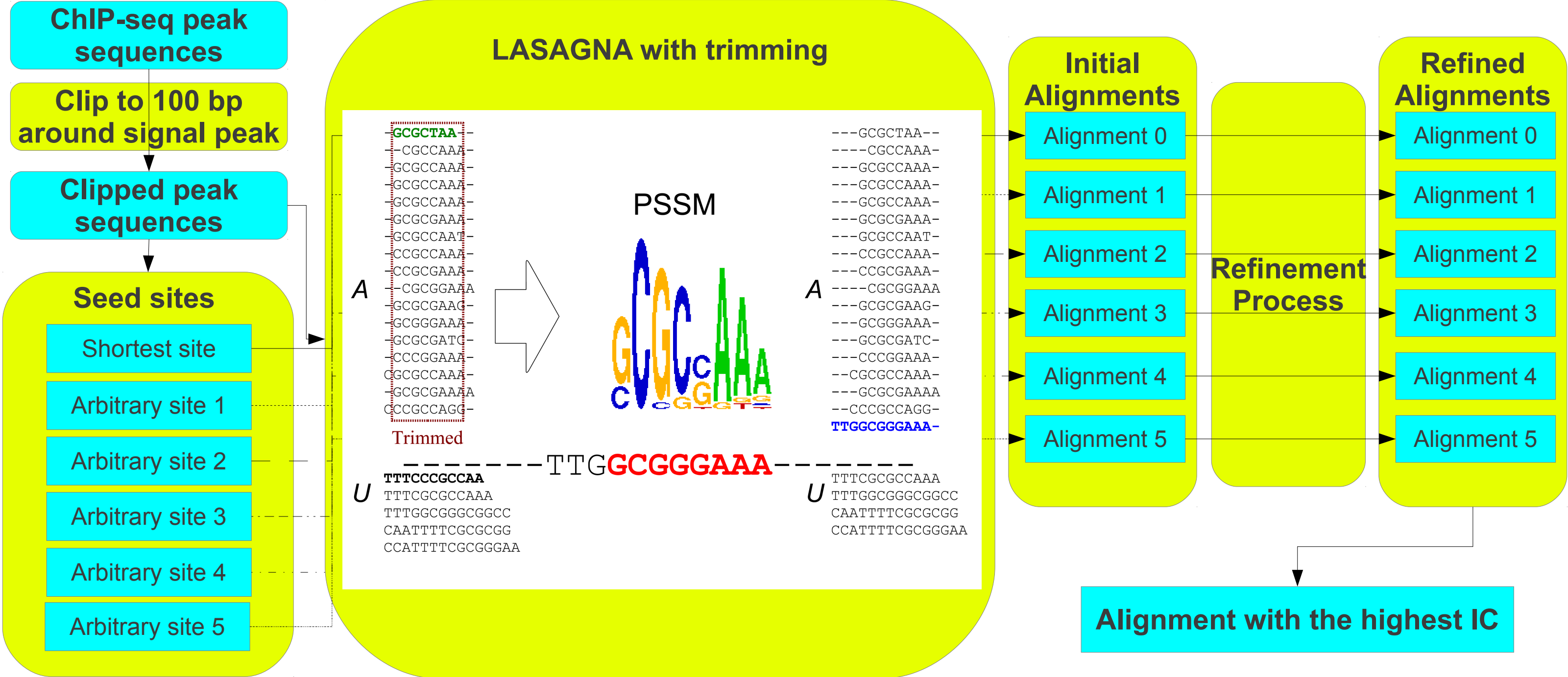

Supplement: Additional file 1 — LASAGNA-ChIP flowchart. [file 1471-2105-14-108-S1.pdf]

LASAGNA

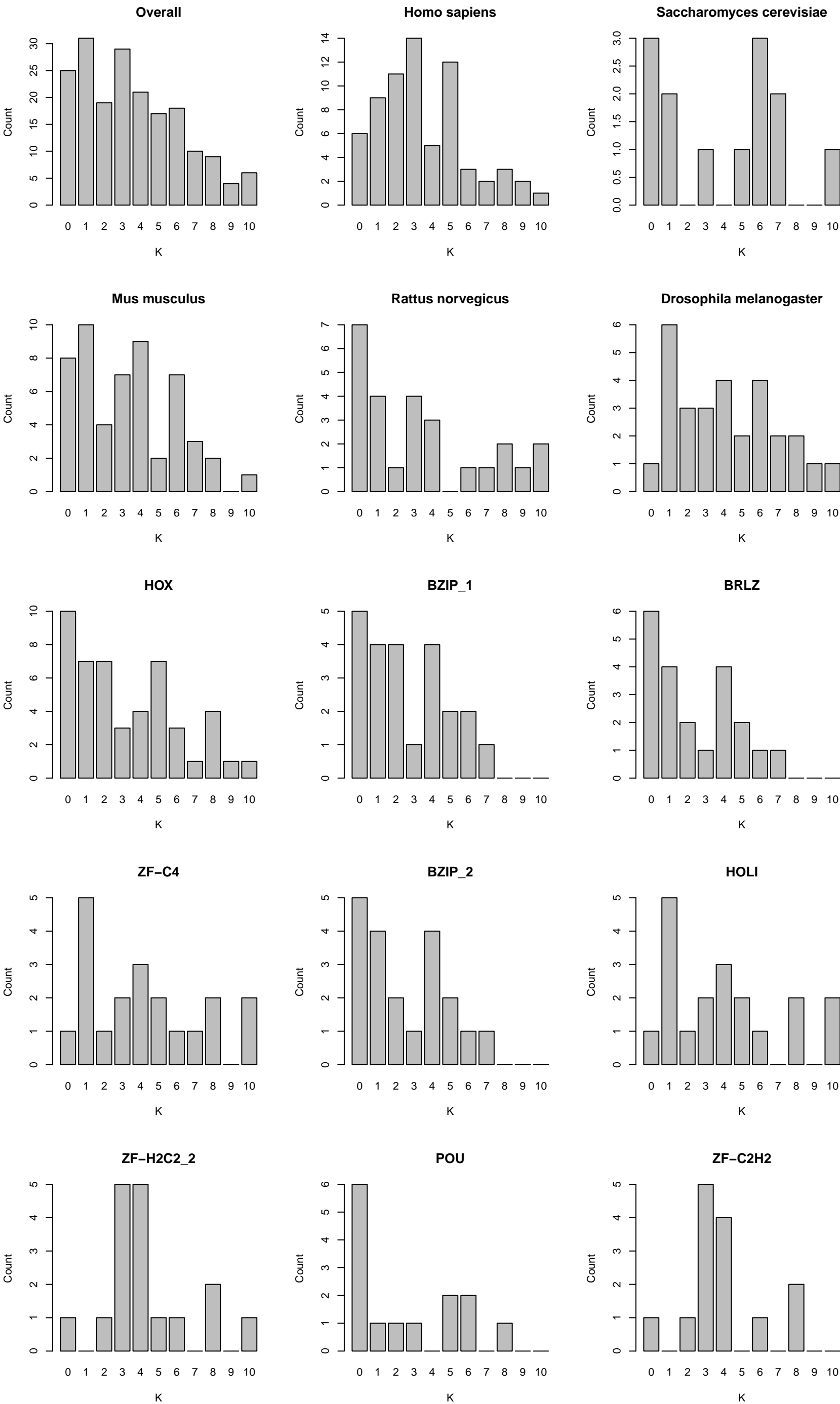

ClustalW2

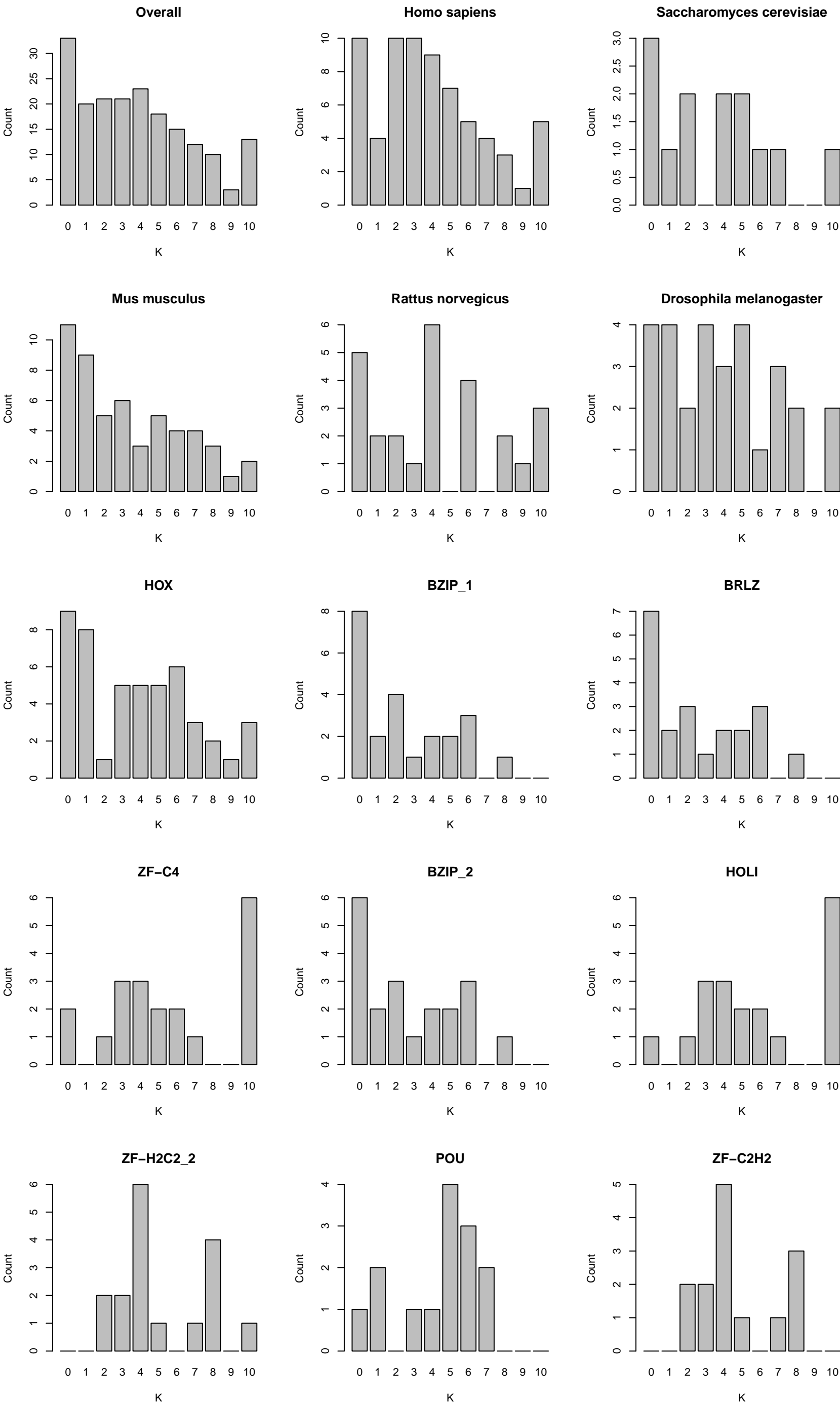

MEME

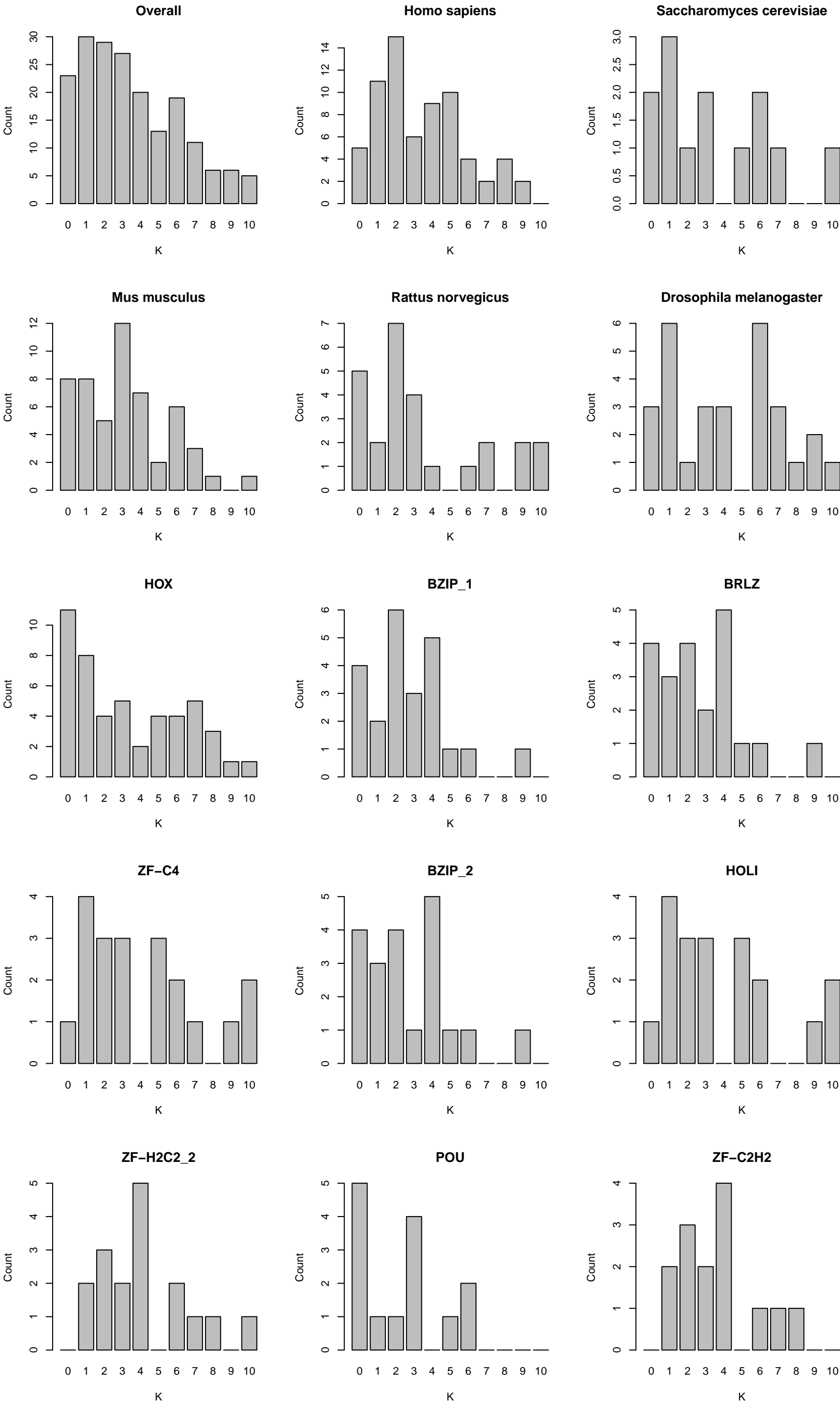

Supplement: Additional file 2 — Distribution of K s by species and conserved domain. [file 1471-2105-14-108-S2.pdf]
