# Supplementary material for: LASAGNA: A novel algorithm for transcription factor binding site alignment
Source: BMC Bioinformatics. 2013 Mar 24;14:108. doi: 10.1186/1471-2105-14-108 (PMC3747862; doi:10.1186/1471-2105-14-108)
Supplement: Additional file 4 — Motifs found by LASAGNA-ChIP and MEME. For each ChIP-seq experiment, the sequence logos of motifs found by LASAGNA-ChIP and MEME are shown. The matching motifs in the TRANSFAC Public and UniPROBE databases found by TOMTOM are listed below each sequence logo. The first ChIPed motif TF is highlighted in yellow if it is among the matching motifs. When the found motif does not resemble those of the ChIPed TF, the first cofactor of the ChIPed TF is highlighted in blue if it is among the matching motifs. Other possibly correct matches are highlighted in green. [file 1471-2105-14-108-S4.pdf]

TF	Cell	LASAGNA-ChIP	MEME
BHLHE40	MEL	wgEncodeSydhTfbsMelBhlhe40cIggrabPk	
		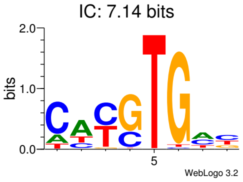 USF; SREBP-1; Arnt; Bhlhb2_primary; N-Myc; c-Myc:Max; Max; PHO4; Max_primary; MyoD; GBP; RAV1; PIF3	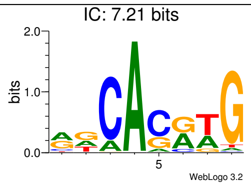 USF; Arnt; N-Myc; SREBP-1; c-Myc:Max; MyoD; Max; Bhlhb2_primary; Max_primary; PHO4; PIF3; Sn; E47; Lmo2complex; GBP; Myf6_primary
BHLHE40	CH12	wgEncodeSydhTfbsCh12Bhlhe40nb100IggrabPk	
		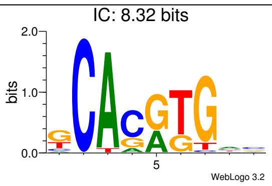 c-Myc:Max; Arnt; USF; Bhlhb2_primary; N-Myc; SREBP-1; Max; PIF3; GBP; Max_primary; Hairy; PHO4; RAV1; Max_secondary; MyoD; Zscan4_primary; Lmo2complex; Tcfe2a_primary; E47	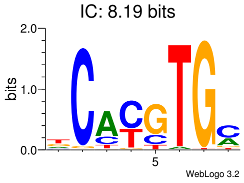 USF; Arnt; c-Myc:Max; Bhlhb2_primary; SREBP-1; Max; Max_primary; N-Myc; PIF3; RAV1; PHO4; Max_secondary; GBP; Hairy; MyoD; Zscan4_primary
CEBPB	C2C12	wgEncodeCaltechTfbsC2c12CebpbFCntrl50bE2p60hPcr1xPkRep1	
		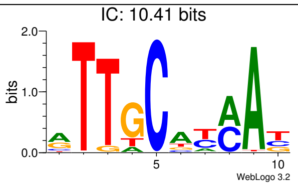 C/EBPbeta; C/EBP; C/EBPalpha; HLF; VBP; E4BP4; Mafb_secondary; CHOP:C/EBPalpha; ces-2; Dlx2_2273.2; Mafk_secondary; Cphx_3484.1; Hdx_3845.3	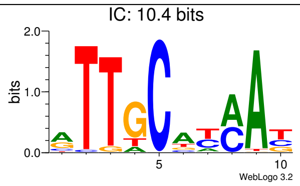 C/EBPbeta; C/EBP; C/EBPalpha; HLF; VBP; E4BP4; Mafb_secondary; CHOP:C/EBPalpha; ces-2; Dlx2_2273.2; Mafk_secondary; Cphx_3484.1
CEBPB	C2C12	wgEncodeCaltechTfbsC2c12CebpbFCntrl50bE2p60hPcr1xPkRep2	
		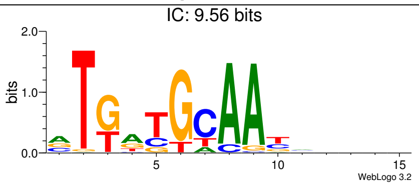 C/EBPbeta; C/EBP; C/EBPalpha; VBP; HLF; CHOP:C/EBPalpha; E4BP4; Mafb_secondary; Zfp105_secondary; MATa1; Dlx2_2273.2; ces-2; Bsx_3483.2; CREB; Oct-1; Cphx_3484.1; Hdx_3845.3; Gmeb1_primary	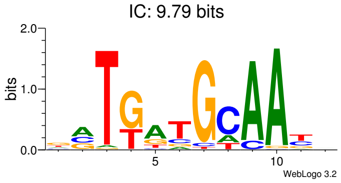 C/EBPbeta; C/EBP; C/EBPalpha; VBP; E4BP4; CHOP:C/EBPalpha; HLF; CREB; MATa1; Mafb_secondary; Cphx_3484.1; ces-2

TF	Cell	LASAGNA-ChIP	MEME
CEBPB	C2C12	wgEncodeCaltechTfbsC2c12CebpbFCntrl50bPcr1xPkRep1	
		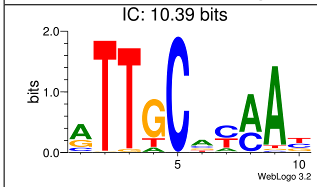 C/EBPbeta; C/EBP; C/EBPalpha; HLF; Mafb_secondary; CHOP:C/EBPalpha; E4BP4; ces-2; VBP; Dlx2_2273.2; Hdx_3845.3; Hoxa6_1040.1; Zfp105_secondary; Mafk_secondary	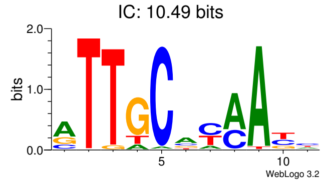 C/EBPbeta; C/EBPalpha; C/EBP; HLF; Mafb_secondary; E4BP4; ces-2; CHOP:C/EBPalpha; VBP; Dlx2_2273.2; Hoxa6_1040.1
JUN	CH12	wgEncodeSydhTfbsCh12CjunIggrabPk	
		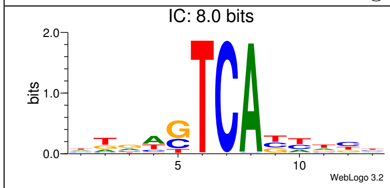 AP-1; GCN4; TCF11:MafG; cap; Jundm2_secondary; TCF11; Bach2; NF-E2; Dfd; CRP; v-Maf; Six4_2860.1	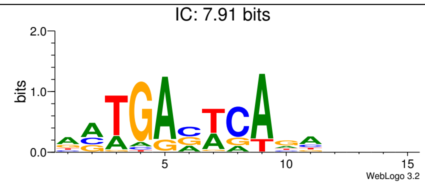 AP-1; Jundm2_secondary; GCN4; NF-E2; TCF11:MafG; v-Maf; Six4_2860.1; Atf1_secondary; Bach2
MYB	MEL	wgEncodeSydhTfbsMelCmybh141IggrabPk	
		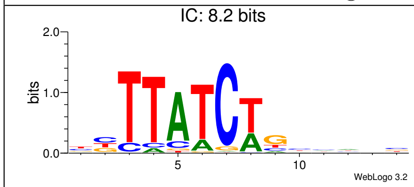 GATA-1; GATA-2; Gata5_primary; Gata6_primary; GATA-6; Gata3_primary ; GATA-3; mtTFA; Lmo2complex; Gata3_secondary; GATA-X; Evi-1; Bbx_secondary; Gabpa_secondary; NIT2	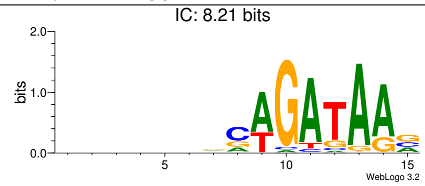 GATA-1; GATA-2; Gata3_primary ; Gata6_primary; Gata5_primary; GATA-3; GATA-6; Lmo2complex; Gata3_secondary; mtTFA; GATA-X; Evi-1; Gabpa_secondary; Sox7_secondary; NIT2
MYB	MEL	wgEncodeSydhTfbsMelCmybsc7874IggrabPk	
		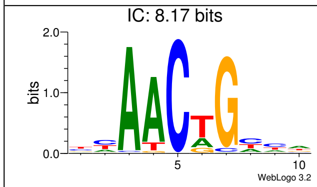 c-Myb; Myb_secondary; Ovo; Mybl1_secondary; v-Myb; GAmyb	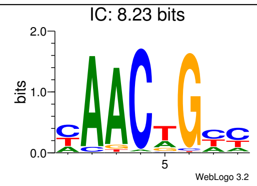 Mybl1_secondary; Myb_secondary; c-Myb; Ovo; GAmyb; v-Myb; MyoD; MIF-1

TF	Cell	LASAGNA-ChIP	MEME
MYC	CH12	wgEncodeSydhTfbsCh12CmycIggrabPk	
		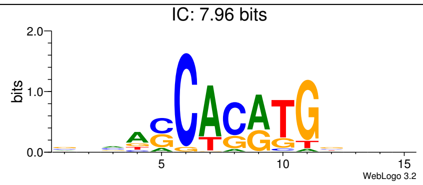 Max_primary; c-Myc:Max; USF; Arnt; Max; Tcfe2a_secondary; N-Myc; Tal-1beta:E47; Lmo2complex; Tal-1alpha:E47; Tal-1beta:ITF-2; PHO4; PIF3; Tcfe2a_primary; Sn; Myf6_primary; Bhlhb2_secondary; E47; Ascl2_primary; MyoD	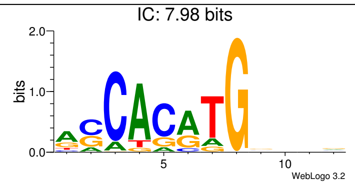 Max_primary; c-Myc:Max; USF; N-Myc; Lmo2complex; PHO4; Max; Arnt; Tcfe2a_secondary; MyoD; Sn; RAV1; PIF3; Tcfe2a_primary; Tal-1alpha:E47; Tal-1beta:E47; E47; GBP; Ascl2_primary; Tal-1beta:ITF-2
MYC	MEL	wgEncodeSydhTfbsMelCmycIggrabPk	
		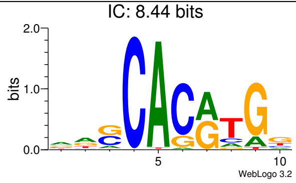 c-Myc:Max; Max_primary; N-Myc; Max; USF; PHO4; Arnt; PIF3; GBP; Lmo2complex; Tcfe2a_secondary; MyoD; Bhlhb2_secondary; Sn; Tcfe2a_primary; Max_secondary; Hairy	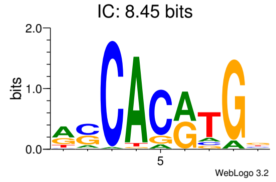 c-Myc:Max; Max_primary; PHO4; Max; N-Myc; USF; Arnt; GBP; PIF3; Lmo2complex; MyoD; Bhlhb2_secondary; Sn; Tcfe2a_secondary
ETS1	CH12	wgEncodeSydhTfbsCh12Ets1IggrabPk	
		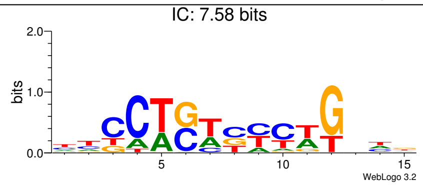 Irf6_secondary; Sox12_secondary; Gabpa_secondary ; Ascl2_secondary; p300; MEIS1; cap	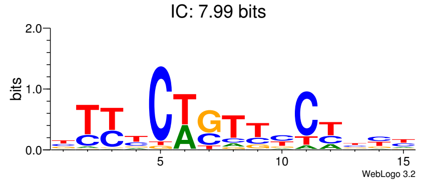 RAV1; cap; Ovo; ISRE; Tcf3_secondary; p300; Sox4_primary
ETS1	MEL	wgEncodeSydhTfbsMelEts1IggrabPk	
		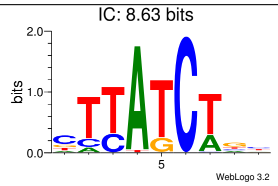 GATA-1; GATA-2; Gata6_primary; GATA-6; GATA-3 ; Gata5_primary; Lmo2complex; mtTFA; Gata3_primary; Evi-1; GATA-X; Gata3_secondary; NIT2	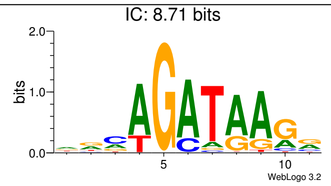 GATA-1; GATA-2; Gata6_primary; Gata5_primary; GATA-6; GATA-3 ; Gata3_primary; GATA-X; mtTFA; Lmo2complex; Evi-1; Gata3_secondary

<table>
<tr><th>TF</th><th>Cell</th><th>LASAGNA-ChIP</th><th>MEME</th></tr>
<tr><td rowspan="3">FOSL1</td><td rowspan="3">C2C12</td><td colspan="2">wgEncodeCaltechTfbsC2c12Fosl1sc605FCntrl36bPcr1xPkRep1</td></tr>
<tr><td>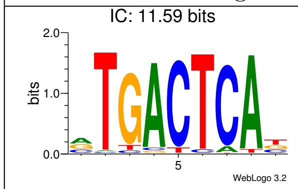
</td><td>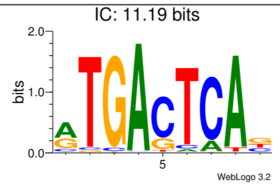
</td></tr>
<tr><td>GCN4; Bach2; AP-1; Bach1; Jundm2_secondary; NF-E2; TCF11:MafG; v-Maf; Tax/CREB; Zfp691_secondary</td><td>AP-1; NF-E2; Bach2; GCN4; Jundm2_secondary; Bach1; TCF11:MafG; v-Maf; Tax/CREB</td></tr>
<tr><td rowspan="3">GATA1</td><td rowspan="3">MEL</td><td colspan="2">wgEncodeSydhTfbsMelGata1Dm2p5dStdPk</td></tr>
<tr><td>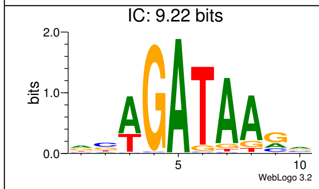
</td><td>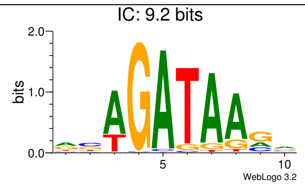
</td></tr>
<tr><td>GATA-2; GATA-1; Gata6_primary; GATA-3; GATA-6; Gata5_primary; Gata3_primary; mtTFA; GATA-X; Lmo2complex; Evi-1; Gata3_secondary; NIT2</td><td>GATA-2; GATA-1; Gata6_primary; GATA-3; GATA-6; Gata3_primary; Gata5_primary; mtTFA; GATA-X; Lmo2complex; Evi-1; NIT2; Gata3_secondary</td></tr>
<tr><td rowspan="3">GATA1</td><td rowspan="3">MEL</td><td colspan="2">wgEncodeSydhTfbsMelGata1IggratPk</td></tr>
<tr><td>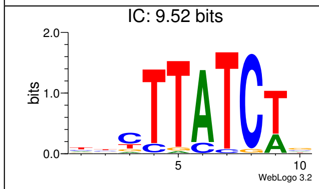
</td><td>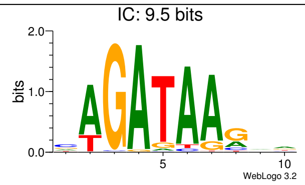
</td></tr>
<tr><td>GATA-1; Gata6_primary; GATA-2; GATA-X; Gata5_primary; Gata3_primary; GATA-3; GATA-6; mtTFA; Lmo2complex; Evi-1; NIT2</td><td>GATA-1; Gata6_primary; GATA-2; GATA-X; Gata3_primary; Gata5_primary; GATA-3; GATA-6; mtTFA; Lmo2complex; Evi-1; NIT2; qa-1F</td></tr>
<tr><td rowspan="3">JUND</td><td rowspan="3">CH12</td><td colspan="2">wgEncodeSydhTfbsCh12JundIggrabPk</td></tr>
<tr><td>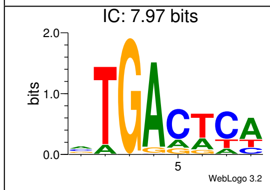
</td><td>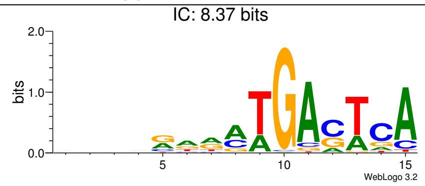
</td></tr>
<tr><td>AP-1; Bach2; TCF11:MafG; GCN4; NF-E2; v-Maf; Bach1; Jundm2_secondary; Mafb_primary; Mafk_primary</td><td>AP-1; GCN4; TCF11:MafG; Jundm2_secondary; Bach2; NF-E2; Bach1; TCF11; v-Maf; cap; Dfd; Zfp691_secondary</td></tr>
</table>

TF	Cell	LASAGNA-ChIP	MEME
JUND	MEL	wgEncodeSydhTfbsMelJundIggrabPk	
		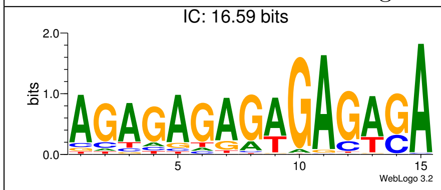<br>p300; Irf6_secondary	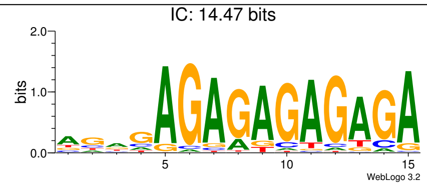<br>Irf6_secondary; p300
MAFK	ES-E14	wgEncodeSydhTfbsEse14MafkStdPk	
		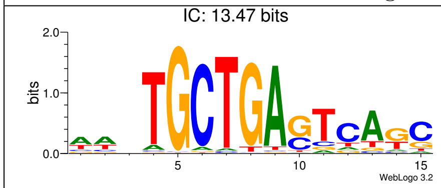<br>v-Maf; TCF11:MafG; NF-E2; Mafb_primary; AP-1; Mafk_primary; Jundm2_secondary; Bach2; GCN4; Bach1; AP-4	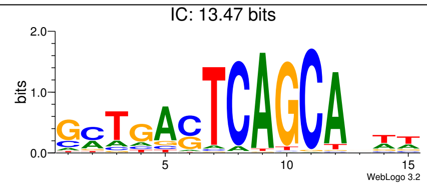<br>v-Maf; TCF11:MafG; NF-E2; Mafb_primary; AP-1; Mafk_primary; Jundm2_secondary; Bach2; GCN4; Bach1; AP-4; XFD-3
MAFK	MEL	wgEncodeSydhTfbsMelMafkDm2p5dStdPk	
		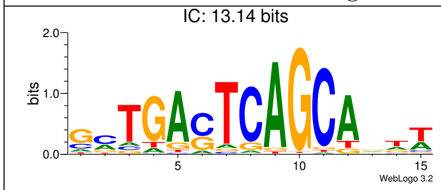<br>v-Maf; TCF11:MafG; NF-E2; Mafb_primary; AP-1; Jundm2_secondary; Mafk_primary; Bach2; GCN4; Bach1; AP-4	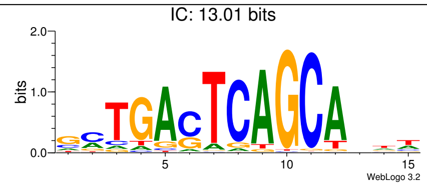<br>v-Maf; TCF11:MafG; NF-E2; AP-1; Mafb_primary; Jundm2_secondary; Mafk_primary; Bach2; GCN4; Bach1; AP-4
MAFK	CH12	wgEncodeSydhTfbsCh12Mafkab50322IggrabPk	
		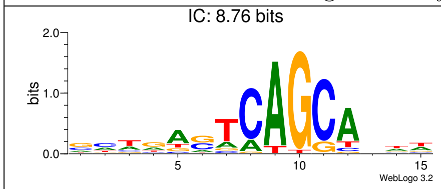<br>v-Maf; Mafb_primary; Mafk_primary; TCF11:MafG; AP-1; NF-E2; Jundm2_secondary; AP-4; XFD-3; RAV1; Zic2_secondary	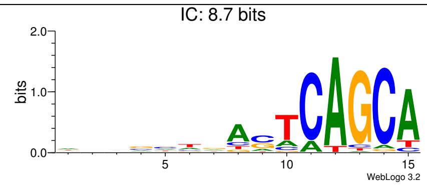<br>v-Maf; Mafb_primary; TCF11:MafG; AP-1; NF-E2; Mafk_primary; Zic2_secondary; Zic1_secondary; Zic3_secondary; Jundm2_secondary; AP-4

TF	Cell	LASAGNA-ChIP	MEME
MAFK	MEL	wgEncodeSydhTfbsMelMafkab50322IggrabPk	
		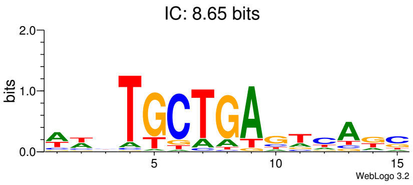 v-Maf; TCF11:MafG; Mafb_primary; NF-E2; AP-1; Mafk_primary; Jundm2_secondary; Bach2; C/EBP	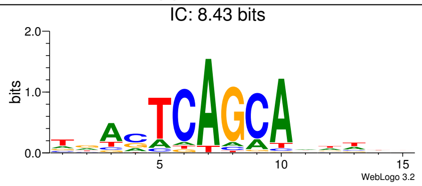 TCF11:MafG; Mafb_primary; Mafk_primary; v-Maf; NF-E2; AP-1; Zic3_secondary; Zic2_secondary; Jundm2_secondary; Zic1_secondary; C/EBP; Pbx1_3203.1; AP-4
MAX	C2C12	wgEncodeCaltechTfbsC2c12MaxFCntrl50bE2p60hPcr1xPkRep1	
		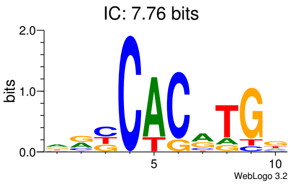 USF; c-Myc:Max; Max; Max_primary; GBP; N-Myc; Arnt; PIF3; PHO4; Tcfe2a_secondary; Max_secondary; Lmo2complex; MyoD; RAV1; Bhlhb2_primary; SREBP-1; Sn	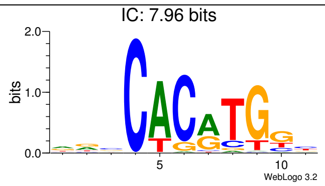 USF; c-Myc:Max; N-Myc; Max; Max_primary; Arnt; GBP; Tcfe2a_secondary; PIF3; PHO4; Lmo2complex; MyoD; Max_secondary; Myf6_primary; RAV1; Tal-1alpha:E47; Tal-1beta:E47; Tcfe2a_primary; Sn; Tal-1beta:ITF-2
MAX	C2C12	wgEncodeCaltechTfbsC2c12MaxFCntrl50bPcr1xPkRep1	
		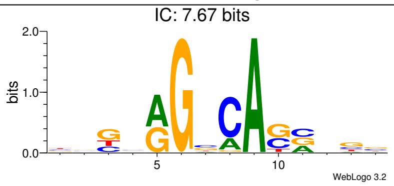 Gabpa_secondary; Hic1_secondary; NF-1; AP-1; Smad3_primary; MyoD; Tcfap2a_secondary; Tcfe2a_secondary; AP-2alpha	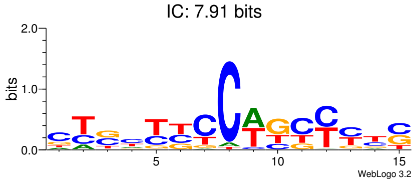 Ascl2_secondary; Gabpa_secondary; cap; Pax-4
MAX	CH12	wgEncodeSydhTfbsCh12MaxIggrabPk	
		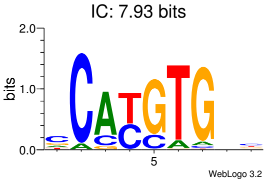 USF; N-Myc; c-Myc:Max; PHO4; Max_primary; Arnt; GBP; Max; PIF3; MyoD; Tcfe2a_secondary; Max_secondary; Lmo2complex; SREBP-1; Bhlhb2_secondary; RAV1	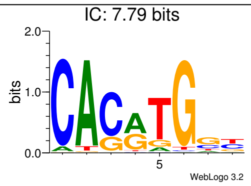 USF; Max_primary; c-Myc:Max; PIF3; N-Myc; Arnt; PHO4; Max; GBP; MyoD; Sn; Lmo2complex; SREBP-1; Tcfe2a_secondary; Zscan4_primary; Max_secondary; Tal-1beta:ITF-2; Tal-1beta:E47; Tal-1alpha:E47; Tcfe2a_primary

TF	Cell	LASAGNA-ChIP	MEME
MAX	MEL	wgEncodeSydhTfbsMelMaxIggrabPk	
		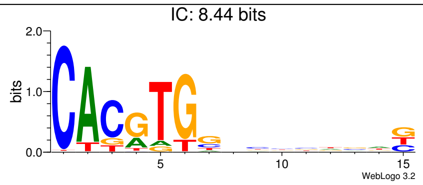 c-Myc:Max; Max_primary; Arnt; USF; PIF3; N-Myc; Max; Bhlhb2_secondary; Bhlhb2_primary; PHO4; GBP; E47; MyoD; Tcfe2a_primary; SREBP-1; HTF; RAV1; Max_secondary; Sn; GR	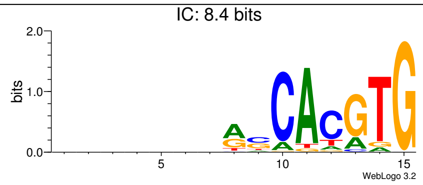 c-Myc:Max; Max_primary; Arnt; Max; Bhlhb2_primary; USF; N-Myc; PHO4; PIF3; Bhlhb2_secondary; GBP; SREBP-1; MyoD; Sn; Max_secondary; Tcfe2a_primary; Myf6_primary; E47; Nkx2-2_2823.1; Hairy
MYOG	C2C12	wgEncodeCaltechTfbsC2c12Sc12732FCntrl32bE2p24hPcr2xPkRep1	
		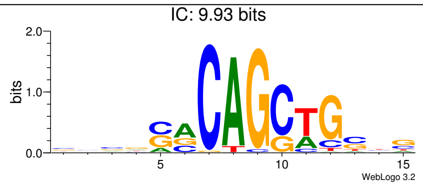 Ascl2_primary; E47; Lmo2complex; AP-4; Myf6_primary; MyoD; Zic1_secondary; Zic2_secondary; Tcfe2a_secondary; Tcfe2a_primary; Zic3_secondary; Sn; HEN1; AREB6; Myf6_secondary; Tgif1_2342.2; Tal-1alpha:E47; RAV1; Tal-1beta:E47; Pknox2_3077.2	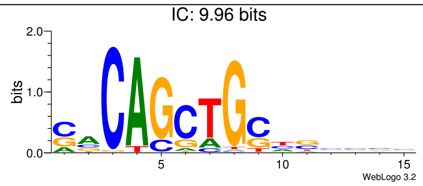 Ascl2_primary; AP-4; Lmo2complex; E47; HEN1; MyoD; Myf6_primary; Tcfe2a_primary; AREB6; Tcfe2a_secondary; Sn; Tgif1_2342.2; Tal-1alpha:E47; Zic1_secondary; Pknox2_3077.2; Zic3_secondary; Gfi-1; Tal-1beta:E47; Zic2_secondary; Tgif2_3451.1
MYOG	C2C12	wgEncodeCaltechTfbsC2c12Sc12732FCntrl32bE2p60hPcr2xPkRep1	
		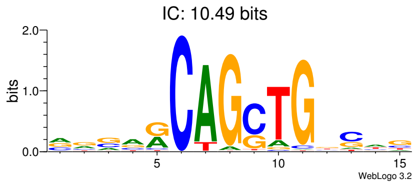 Myf6_primary; Ascl2_primary; E47; AP-4; MyoD; Tcfe2a_secondary; Tcfe2a_primary; Lmo2complex; HEN1; Sn; Tal-1beta:E47; Tal-1beta:ITF-2; AREB6; RAV1; Tal-1alpha:E47; c-Myc:Max; USF; Tgif1_2342.2; myogenin/NF-1; Mybl1_secondary	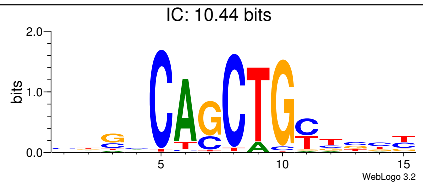 Myf6_primary; Ascl2_primary; E47; AP-4; Lmo2complex; MyoD; Tcfe2a_primary; HEN1; Tcfe2a_secondary; Sn; Tal-1beta:E47; Tal-1beta:ITF-2; Tal-1alpha:E47; AREB6; RAV1; USF; Tgif1_2342.2; c-Myc:Max; Arnt; myogenin/NF-1
MYOG	C2C12	wgEncodeCaltechTfbsC2c12Sc12732FCntrl50bE2p7dPcr1xPkRep1	
		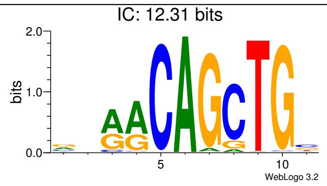 Ascl2_primary; Myf6_primary; Sn; MyoD; AP-4; HEN1; E47; Lmo2complex; Tcfe2a_secondary; Tal-1beta:ITF-2; myogenin/NF-1; Tal-1alpha:E47; RP58; Tcfe2a_primary; Myf6_secondary; Tgif1_2342.2; Tal-1beta:E47; AREB6; Tgif2_3451.1; Pknox2_3077.2	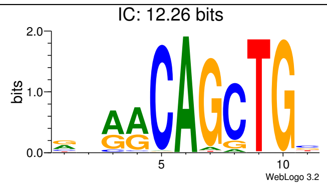 Ascl2_primary; Myf6_primary; Sn; MyoD; AP-4; HEN1; E47; Lmo2complex; Tcfe2a_secondary; myogenin/NF-1; Tal-1beta:ITF-2; Tal-1alpha:E47; RP58; Tcfe2a_primary; Tal-1beta:E47; Myf6_secondary; Tgif1_2342.2; AREB6; Pknox2_3077.2; Tgif2_3451.1

TF	Cell	LASAGNA-ChIP	MEME
MYOD1	C2C12	wgEncodeCaltechTfbsC2c12Sc32758FCntrl32bE2p24hPcr2xPkRep1	
		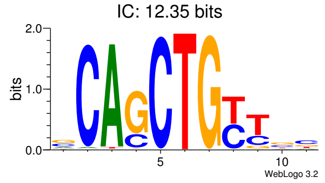 Ascl2_primary; Myf6_primary; MyoD; HEN1; Sn; AP-4; Lmo2complex; E47; Tcfe2a_primary; Tal-1beta:ITF-2; Tal-1alpha:E47; myogenin/NF-1; Tal-1beta:E47; RP58; Tcfe2a_secondary; Myf6_secondary; RAV1; AREB6; Tgif1_2342.2; Adf-1	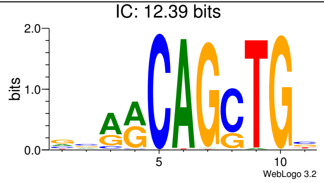 Ascl2_primary; Myf6_primary; MyoD; Sn; HEN1; AP-4; E47; Lmo2complex; Tcfe2a_primary; Tal-1beta:ITF-2; Tal-1alpha:E47; myogenin/NF-1; Tcfe2a_secondary; RP58; Tal-1beta:E47; Myf6_secondary; AREB6; RAV1; Tgif1_2342.2; Pknox2_3077.2
MYOD1	C2C12	wgEncodeCaltechTfbsC2c12Sc32758FCntrl32bE2p60hPcr2xPkRep1	
		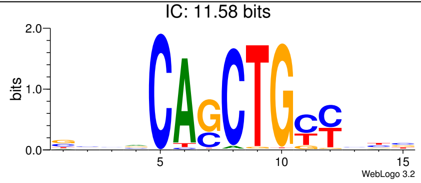 Ascl2_primary; Myf6_primary; MyoD; E47; Lmo2complex; Sn; AP-4; Tcfe2a_primary; HEN1; Tcfe2a_secondary; Mybl1_secondary; Myb_secondary; Tal-1beta:ITF-2; Tal-1alpha:E47; Tal-1beta:E47; Myf6_secondary; RAV1; c-Myb; c-Myc:Max; AREB6	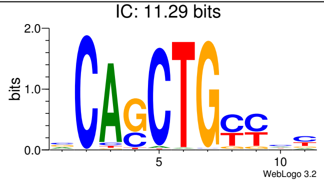 Ascl2_primary; MyoD; Myf6_primary; Sn; HEN1; E47; AP-4; Lmo2complex; Tcfe2a_secondary; Tcfe2a_primary; RAV1; Tal-1alpha:E47; Tgif1_2342.2; Tal-1beta:ITF-2; myogenin/NF-1; AREB6; Myb_secondary; Tal-1beta:E47; USF; c-Myb
MYOD1	C2C12	wgEncodeCaltechTfbsC2c12Sc32758FCntrl32bPcr2xPkRep1	
		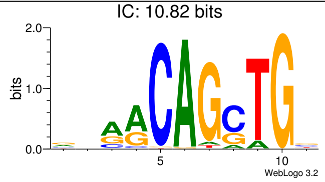 Myf6_primary; Ascl2_primary; MyoD; Sn; E47; AP-4; HEN1; Tal-1beta:ITF-2; Tal-1alpha:E47; Lmo2complex; myogenin/NF-1; Tcfe2a_primary; Tal-1beta:E47; Tcfe2a_secondary; RP58; AREB6; Myf6_secondary; RAV1; Tgif1_2342.2; USF	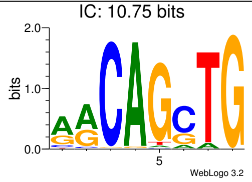 Ascl2_primary; Myf6_primary; MyoD; AP-4; E47; Sn; myogenin/NF-1; Tal-1alpha:E47; Tcfe2a_primary; Lmo2complex; HEN1; Tgif2_3451.1; Tgif1_2342.2; Tal-1beta:ITF-2; Pknox2_3077.2; Tal-1beta:E47; Mrg1_2246.2; Meis1_2335.1; AREB6; Mrg2_2302.1
MYOD1	C2C12	wgEncodeCaltechTfbsC2c12Sc32758FCntrl50bE2p7dPcr1xPkRep1	
		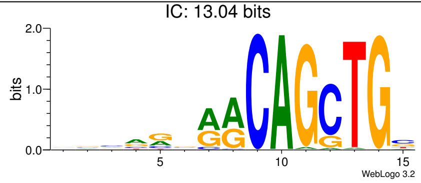 Ascl2_primary; Myf6_primary; HEN1; MyoD; Sn; AP-4; E47; Lmo2complex; Tcfe2a_secondary; Tgif1_2342.2; Tcfe2a_primary; Myf6_secondary; c-Myb; Tal-1beta:ITF-2; Pknox2_3077.2; RP58; Tal-1alpha:E47; Tal-1beta:E47; Eomes_secondary; AREB6	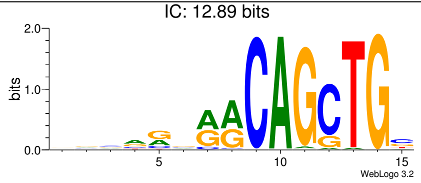 Ascl2_primary; Myf6_primary; HEN1; MyoD; Sn; AP-4; E47; Tcfe2a_secondary; Lmo2complex; Tgif1_2342.2; Tcfe2a_primary; c-Myb; Myf6_secondary; Pknox2_3077.2; RP58; Tal-1beta:ITF-2; Tal-1alpha:E47; Tal-1beta:E47; AREB6; myogenin/NF-1

TF	Cell	LASAGNA-ChIP	MEME
SRF	C2C12	wgEncodeCaltechTfbsC2c12SrfFCntrl32bE2p24hPcr2xPkRep1	
		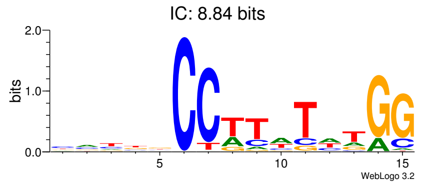 SRF; Srf_primary; AG; YY1; AGL3; TATA; Tbp_secondary; MCM1; GATA-1; Msx-1	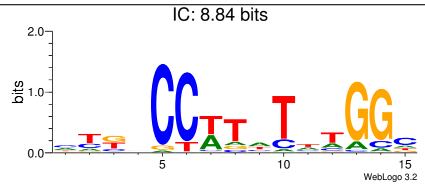 SRF; Srf_primary; AG; AGL3; YY1; MCM1; Tbp_secondary; TATA; GATA-1; Abd-B; Tcf3_secondary
TBP	CH12	wgEncodeSydhTfbsCh12TbpIggmusPk	
		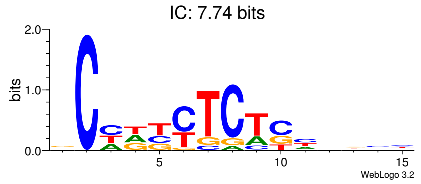 Gabpa_secondary; Sox12_secondary	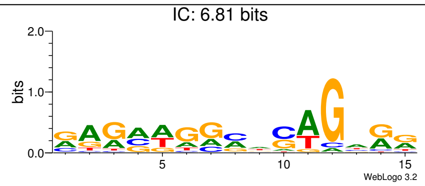 Pax-4; cap; Gabpa_secondary; Irf3_secondary
TBP	MEL	wgEncodeSydhTfbsMelTbpIggmusPk	
		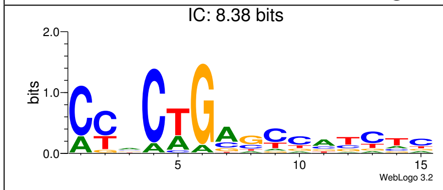 AP-2rep; CDC5; AP-1; Pax-4; NF-E2; RAV1	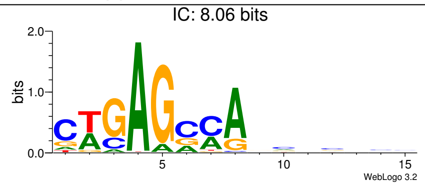 CDC5; Gfi-1; AP-1; GCN4; v-Maf; TCF11:MafG; myogenin/NF-1
TCF3	C2C12	wgEncodeCaltechTfbsC2c12Tcf3FCntrl32bE2p5dPcr2xPkRep1	
		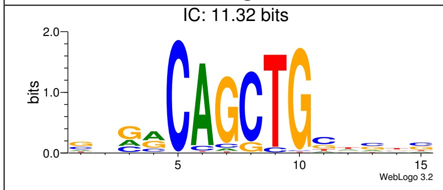 Ascl2_primary; Myf6_primary; MyoD; E47; Lmo2complex; AP-4; Sn; Tcfe2a_secondary; Tcfe2a_primary; Tal-1alpha:E47; HEN1; Tal-1beta:ITF-2; Tal-1beta:E47; Myf6_secondary; Myb_secondary; Tgif1_2342.2; AREB6; Mybl1_secondary; RAV1; Arnt	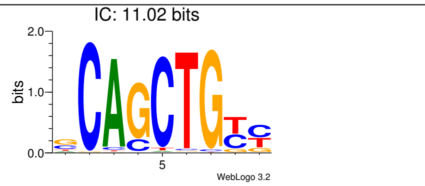 Ascl2_primary; MyoD; AP-4; Tgif1_2342.2; HEN1; Lmo2complex; Myf6_primary; Sn; Tgif2_3451.1; Meis1_2335.1; Pknox2_3077.2; Tcfe2a_primary; Mrg1_2246.2; E47; Mrg2_2302.1; myogenin/NF-1; Tcfe2a_secondary; Pknox1_2364.2; TGIF; AREB6

<table>
<tr><th>TF</th><th>Cell</th><th>LASAGNA-ChIP</th><th>MEME</th></tr>
<tr><td rowspan="2">USF1</td><td rowspan="2">C2C12</td><td colspan="2">wgEncodeCaltechTfbsC2c12Usf1FCntrl50bE2p60hPcr1xPkRep1</td></tr>
<tr><td>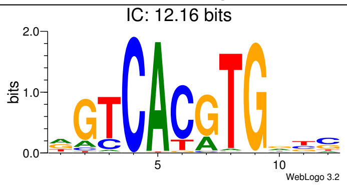

USF; Arnt; SREBP-1; Bhlhb2_primary; c-Myc:Max; Max; GBP; N-Myc; PIF3; Max_secondary; Max_primary; RAV1; XBP-1; Hairy; PHO4; MyoD; bZIP911; Rara_primary; ATF6; AREB6</td><td>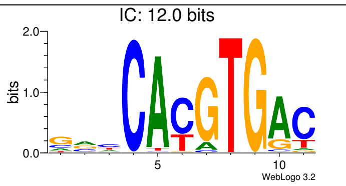

USF; Arnt; SREBP-1; Bhlhb2_primary; GBP; c-Myc:Max; PIF3; Max; N-Myc; Max_secondary; Max_primary; RAV1; bZIP911; XBP-1; PHO4; MyoD; Hairy; Rara_primary; ATF6; CF1/USP</td></tr>
<tr><td rowspan="2">USF1</td><td rowspan="2">C2C12</td><td colspan="2">wgEncodeCaltechTfbsC2c12Usf1FCntrl50bPcr1xPkRep1</td></tr>
<tr><td>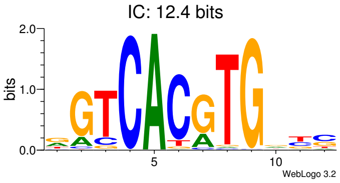

USF; Arnt; SREBP-1; Bhlhb2_primary; c-Myc:Max; Max; GBP; N-Myc; PIF3; Max_secondary; Max_primary; XBP-1; RAV1; Hairy; bZIP911; Rara_primary; MyoD; Rxra_primary; PHO4; CF1/USP</td><td>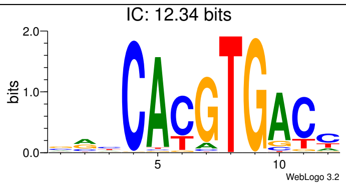

USF; Arnt; Bhlhb2_primary; SREBP-1; c-Myc:Max; Max; GBP; N-Myc; PIF3; Max_secondary; Max_primary; XBP-1; Rara_primary; RAV1; bZIP911; Hairy; Rxra_primary; CF1/USP; Nr2f2_primary; MyoD</td></tr>
</table>
